# Supplementary material for: New Insights into the Medieval Hispano-Muslim Panel Painting: The Alfarje Found in a Balearic Casal (Spain)
Source: Molecules. 2023 Jan 27;28(3):1235. doi: 10.3390/molecules28031235 (PMC9921033; doi:10.3390/molecules28031235)
Supplement: Supplementary file 1 [file molecules-28-01235-s001.zip › molecules-2160582-supplementary.pdf]

Table S1.- Main morphological properties found in the set of samples analysed using optical microscopy.

**C-2**			
**Microphotograph of the fragment**	**Microphotograph of the cross-section (oblique illumination)**	**Microphotograph of the cross-section (PPL)**	**Microphotograph of the cross-section (XPL)**
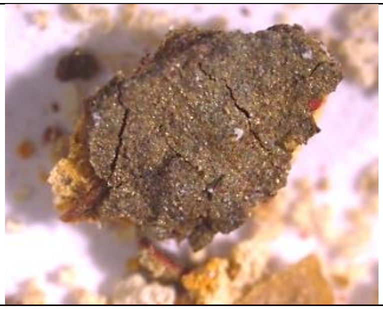	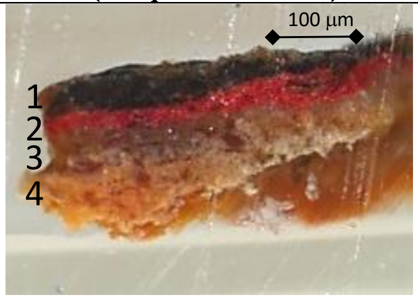	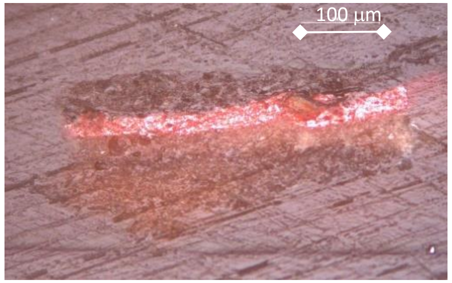	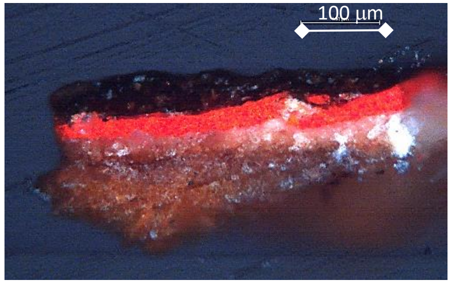
**Layer assignment**	**Average thickness of layers (µm)**	**Morphological and optical properties**	
**Surface:** Alteration materials<br>**1.** Protective coating<br>**2.** Paint layer<br>**3.** Upper ground layer<br>**4.** Inner ground layer	**1.** 53<br>**2.** 20<br>**3.** 40<br>**4.** >80	**Surface:** Abundant cryptocrystalline greyish aggregates deposited on the surface of the paint<br>**1.** and brownish translucent layer with abundant nodules of opaque matter dispersed in the amorphous matrix of the varnish<br>**2.** Red layer composed of coarse grains of cinnabar that exhibits birefringence. The particles of pigment have irregular shape and present a size distribution ranging from coarse to very fine.<br>**3.** Off white upper ground layer with abundant binding medium that obscures the pigment.<br>**4.** Ground made with very coarse to fine aggregates of off white pigment with scattered impurities due to the presence of abundant darkened binding medium.	

<table>
<tr><th colspan="4">C-4</th></tr>
<tr><th>Microphotograph of the fragment</th><th>Microphotograph of the cross-section (oblique illumination)</th><th>Microphotograph of the cross-section (PPL)</th><th>Microphotograph of the cross-section (XPL)</th></tr>
<tr><td>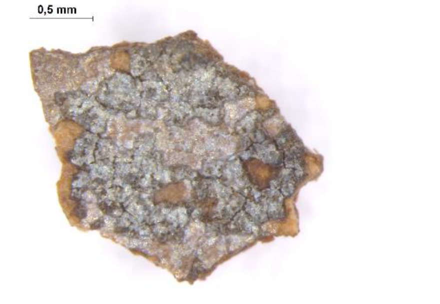
</td><td>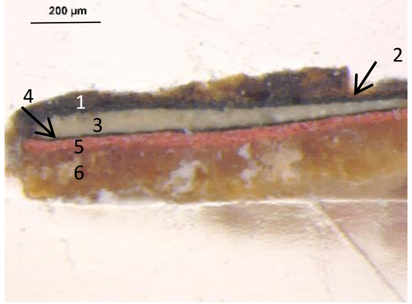
</td><td>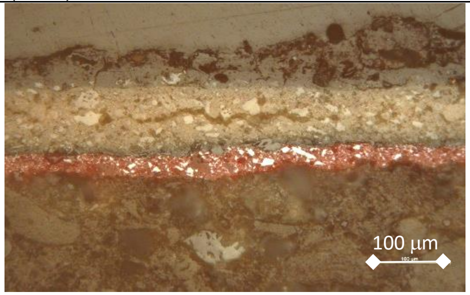
</td><td>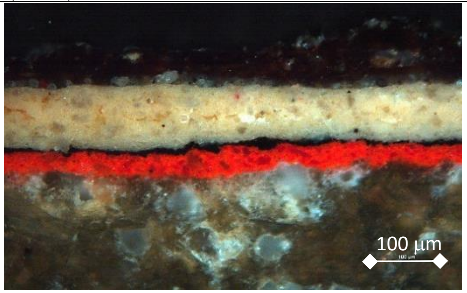
</td></tr>
<tr><th>Layer assignment</th><th>Layer thickness (µm)</th><th colspan="2">Morphological and optical properties</th></tr>
<tr><td>Surface: Altered upper part of the coating layer
1. Lower part of the coating layer
2. Paint layer
3. Thin organic layer
4. Paint layer
5. Ground</td><td>Surface: 31
1.19
2. 45
3. 8
4. 29
5. >120</td><td colspan="2">Surface: Abundant cryptocrystalline greyish aggregates protruding on the surface of the coating varnish
1 and 2. Amorphous and brownish translucent organic material with some coarse white grains of pigment
3. White layer. This pigment presents a particle size distribution ranging from coarse to very fine grain size. The particles have irregular shapes.
4. Amorphous and brownish translucent thin organic layer
5. Red layer composed of coarse grains of cinnabar that exhibits birefringence. This pigment presents size distribution of grains ranging from coarse to very fine size. The particles have elongated and irregular shapes
6. Ground made with very coarse to fine aggregates of off white pigment with scattered impurities due to the presence of abundant darkened binding medium.</td></tr>
</table>

C-5			
**Microphotograph of the fragment**	**Microphotograph of the cross-section (oblique illumination)**	**Microphotograph of the cross-section (PPL)**	**Microphotograph of the cross-section (XPL)**
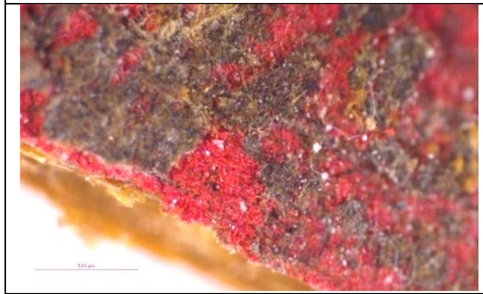	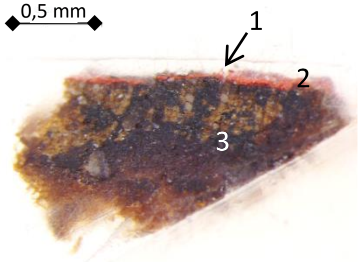	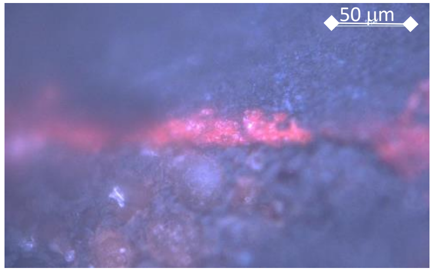	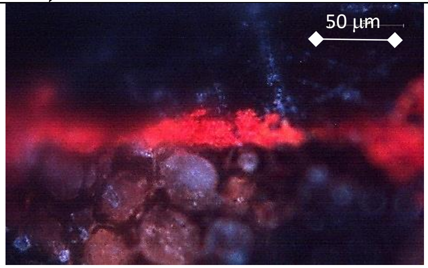
**Layer assignment**	**Layer thickness (µm)**	**Morphological and optical properties**	
**Surface:** Alteration materials embedded in a highly degraded coating layer<br>**1.** Upper paint layer<br>**2**. Inner paint layer<br>**3.** Support	**1.** n.m.<br>**2.** 30<br>**3.** n.m.	**Surface.** A network of hyphae comprised of long filamentous hypha of fungi form a network on the surface of the coating<br>**1.** Red layer composed of coarse grains of cinnabar that exhibits characteristic birefringence. This pigment presents a size distribution of grains ranging from coarse to very fine size. The particles have irregular shapes<br>**2.** Wood support impregnated in its upper part with white. The wood exhibits anatomic elements characteristic of a coniferae in radial section, The cells appear filled with white pigment near the paint layers	

n.m.: not measured

D-1			
**Microphotograph of the fragment**	**Microphotograph of the cross-section (oblique illumination)**	**Microphotograph of the cross-section (PPL)**	**Microphotograph of the cross-section (XPL)**
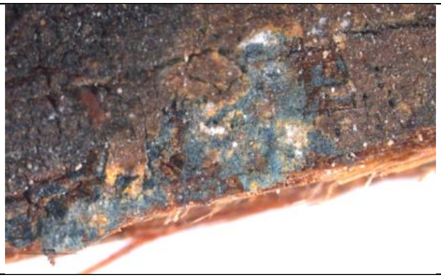	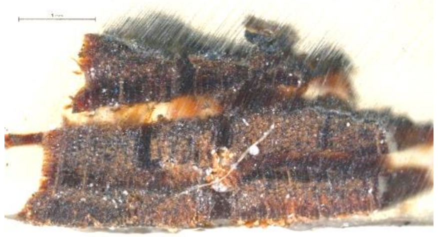	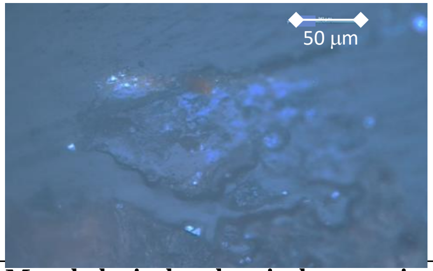	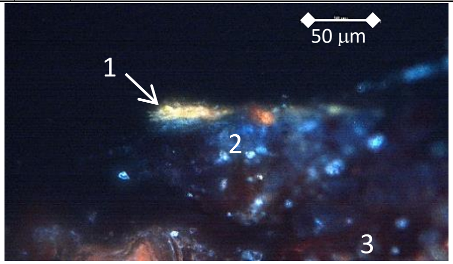
**Layer assignment**	**Layer thickness (µm)**	**Morphological and optical properties**	
**Surface:** Alteration materials<br>**1.** Top paint layer<br>**2.** Lower paint layer<br>**3.** Support	**1.** 10<br>**2.** 50<br>**3.** n.m.	**Surface:** A network of hyphae comprised of long filamentous hypha of fungi form a network on the surface of the paint<br>**1** Yellow layer.<br>**2.** Blue layer<br>**3.** Wood support impregnated in its upper part with white. The wood exhibits anatomic elements characteristic of a coniferae in radial section, The cells appear filled with white pigment near the paint layers	

<table>
<tr><th colspan="4">F-1</th></tr>
<tr><th>Microphotograph of the fragment</th><th>Microphotograph of the cross-section (oblique illumination)</th><th>Microphotograph of the cross-section (PPL)</th><th>Microphotograph of the cross-section (XPL)</th></tr>
<tr><td>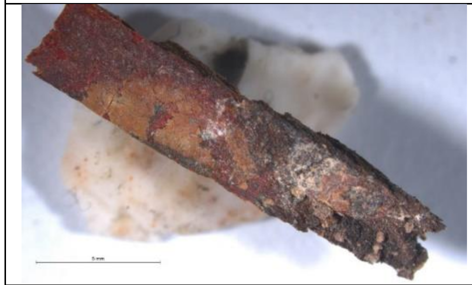</td><td>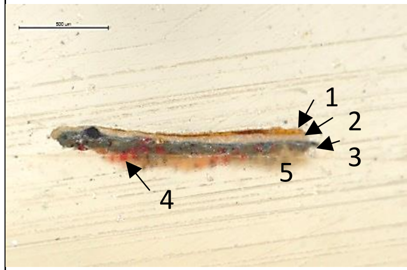</td><td>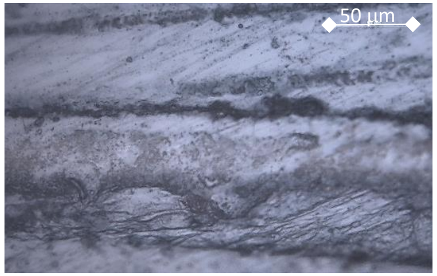</td><td>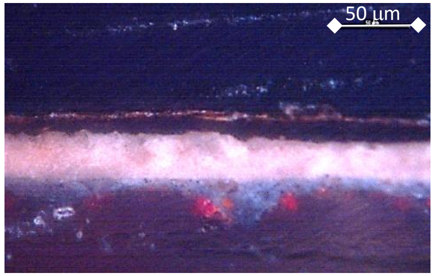</td></tr>
<tr><th>Layer assignment</th><th>Layer thickness (µm)</th><th colspan="2">Morphological and optical properties</th></tr>
<tr><td>Surface: Alteration materials
1. Protective coating
2. Top paint layer
3. Middle paint layer
4. Bottom paint layer
5. Ground</td><td>1. 9
2. 15
3. 20
4. 28
5. -</td><td colspan="2">Surface: Cryptocrystalline materials formed or deposited on the surface of the paint
1 Amorphous and brownish translucent layer
2. White layer. This pigment presents a size distribution of grains ranging from coarse to very fine. The particles have irregular shapes.
3. Light blue layer comprising coarse to fine grains of white pigment and fine to very fine grains of a dark blue pigment.
4. Layer formed with red particles that exhibits characteristic birefringence of cinnabar.
5. Ground made with very coarse to fine aggregates of off white pigment with scattered impurities due to the presence of abundant darkened binding medium.</td></tr>
</table>

<table>
<tr><th colspan="4">F-4</th></tr>
<tr><th>Microphotograph of the fragment</th><th>Microphotograph of the cross-section (oblique illumination)</th><th>Microphotograph of the cross-section (PPL)</th><th>Microphotograph of the cross-section (XPL)</th></tr>
<tr><td>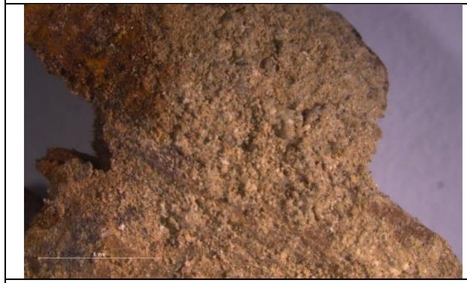</td><td>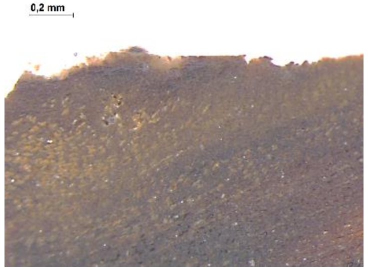
</td><td>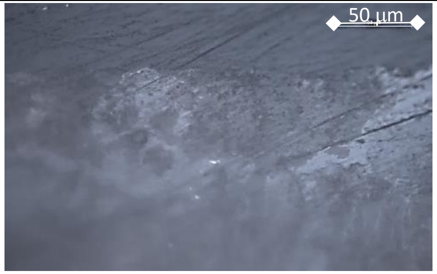
</td><td>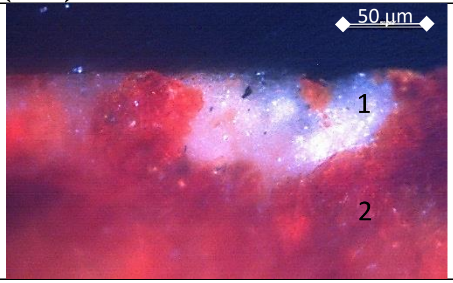
</td></tr>
<tr><th>Layer assignment</th><th>Layer thickness (µm)</th><th colspan="2">Morphological and optical properties</th></tr>
<tr><td>Surface: Alteration materials
1. Paint layer
2. Support</td><td>1. 77
2. n.m.</td><td colspan="2">Surface: Cryptocrystalline deposits of clayey minerals ranging in color from dark to pale yellow with accessory minerals of different colors. The size distribution ranging from coarse to very fine. The particles have irregular shapes.
1. Blue-white layer
2. Wood support impregnated in its upper part with white. The wood exhibits anatomic elements characteristic of a coniferae in transversal section, The cells appear filled with white pigment near the paint layers</td></tr>
</table>

n.m.: not measured

<table>
<tr><th colspan="4">J-2</th></tr>
<tr><th>Microphotograph of the fragment</th><th>Microphotograph of the cross-section (oblique illumination)</th><th>Microphotograph of the cross-section (PPL)</th><th>Microphotograph of the cross-section (XPL)</th></tr>
<tr><td>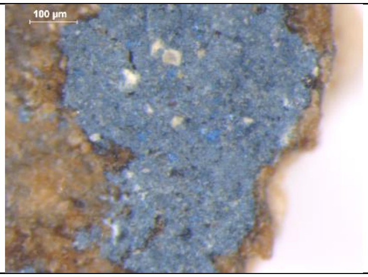
</td><td>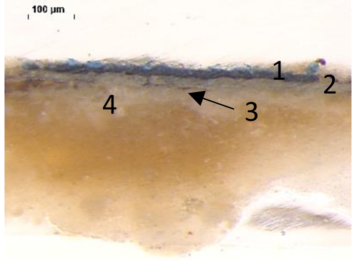
</td><td>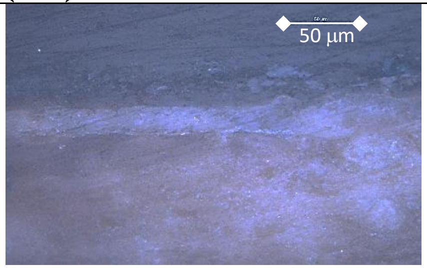
</td><td>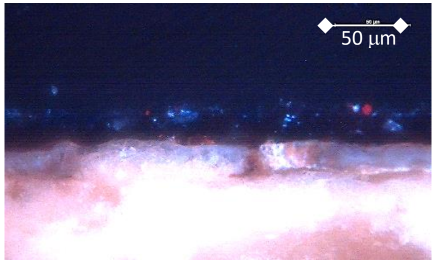
</td></tr>
<tr><th>Layer assignment</th><th>Layer thickness (µm)</th><th colspan="2">Morphological and optical properties</th></tr>
<tr><td>Surface: Alteration materials
1. Top paint layer
2. Thin metal foil
3. Altered metal foil
4. Ground</td><td>1. 25
2. 25
3.12 -37
4. > 300</td><td colspan="2">Surface: Cryptocrystalline materials formed or deposited on the surface of the paint
1. Blue-white layer. The particles exhibit blue hue ranging from deep blue to greenish-blue hue. The grains have irregular shape and a size distribution ranging from coarse to fine size. Observed with polarized light the particles exhibit pleochroism typical of azurite.
2. Thin layer of powdered laminar metal.
3. Reticulated layer of altered metal
4. Ground made with very coarse to fine aggregates of off white pigment with scattered impurities due to the presence of abundant darkened binding medium.</td></tr>
</table>

J-3			
**Microphotograph of the fragment**	**Microphotograph of the cross-section (oblique illumination)**	**Microphotograph of the cross-section (PPL)**	**Microphotograph of the cross-section (XPL)**
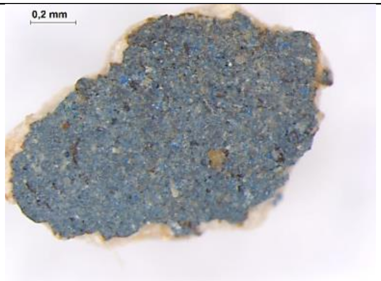	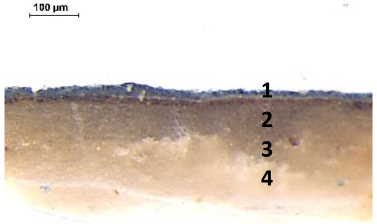	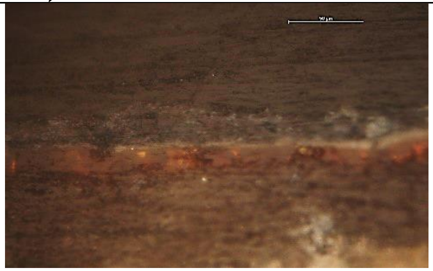	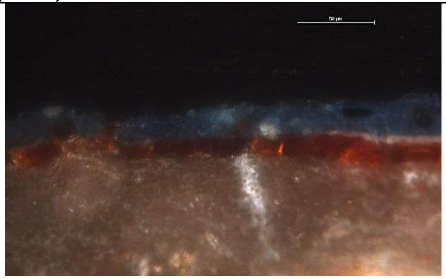
**Layer assignment**	**Layer thickness (µm)**	**Morphological and optical properties**	
**Surface:** Alteration materials<br>**1.** Top paint layer<br>**2.** Middle paint layer<br>**3.** Bottom paint layer<br>**4.** Ground	**1.** 25<br>**2.** 4<br>**3.** 12<br>**4.** 190	**Surface:** Cryptocrystalline materials formed or deposited on the surface of the paint<br>**1.** Blue layer. The particles exhibit blue hue ranging from deep blue to greenish-blue hue. The grains have irregular shape and a size distribution ranging from coarse to fine size. Observed with polarized light the particles exhibit pleochroism typical of azurite.<br>**2.** White layer. This pigment presents size distribution ranging from coarse to fine size. The particles have irregular shapes.<br>**3.** Amorphous brownish translucent layer<br>**4.** Ground made with very coarse to fine aggregates of off white pigment with scattered impurities due to the presence of abundant darkened binding medium.	

J-4			
**Microphotograph of the fragment**	**Microphotograph of the cross-section (oblique illumination)**	**Microphotograph of the cross-section (PPL)**	**Microphotograph of the cross-section (XPL)**
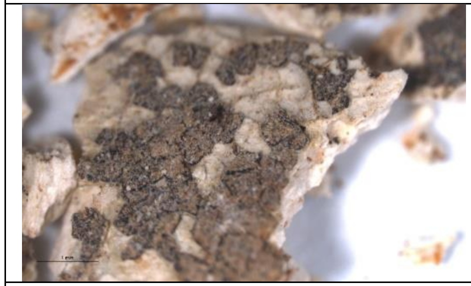	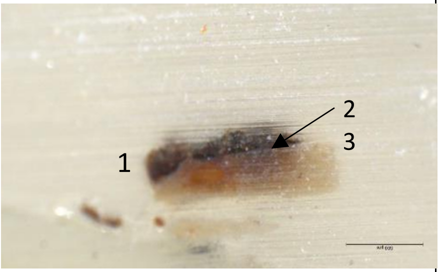	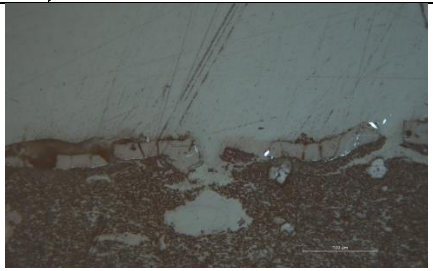	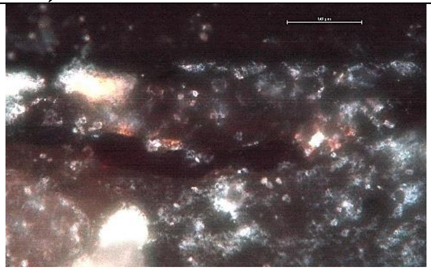
**Layer assignment**	**Layer thickness (μm)**	**Morphological and optical properties**	
**Surface:** Alteration materials<br>**1.** Organic coating layer<br>**2.** Metal foil<br>**3.** Ground	**1.** 20<br>**2.** n.m.<br>**3.** >200	**Surface:** Cryptocrystalline aggregates of clayey materials<br>**1.** Amorphous and brownish translucent layer<br>**2.** Thin foil of white metal probably silver<br>**3.** Ground made with very coarse to fine aggregates of off white pigment with scattered impurities due to the presence of abundant darkened binding medium.	

n.m.: not measured

K-4			
**Microphotograph of the fragment**	**Microphotograph of the cross-section (oblique illumination)**	**Microphotograph of the cross-section (PPL)**	**Microphotograph of the cross-section (XPL)**
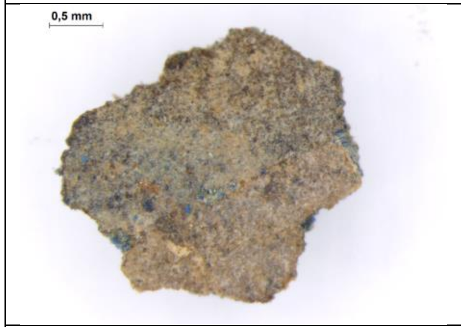	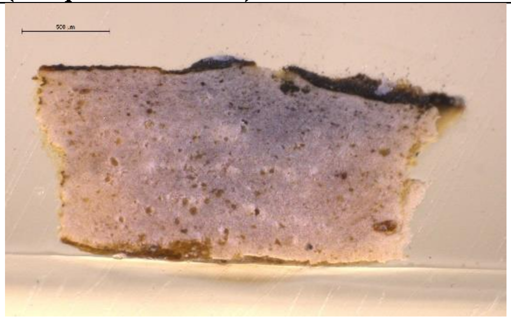	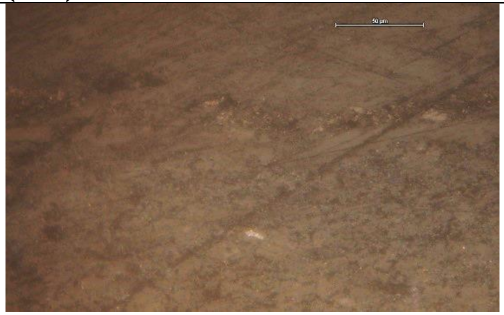	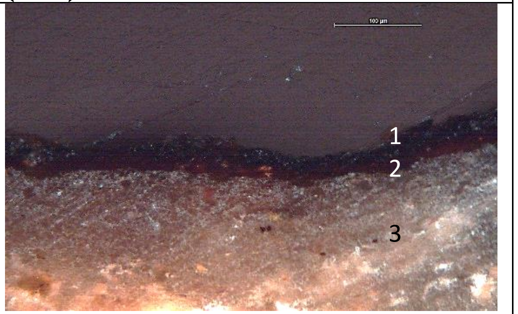
**Layer assignment**	**Layer thickness (μm)**	**Morphological and optical properties**	
**Surface:** Alteration materials<br>**1.** Paint layer<br>**2.** Organic layer<br>**3.** Ground	**1.** 40<br>**2.** 15<br>**3.** >664	**Surface**: Cryptocrystalline white aggregates formed or deposited on the surface of the painting<br>**1.** Blue layer. The particles exhibit blue hue ranging from deep blue to greenish-blue hue. The grains have irregular shape and a size distribution ranging from coarse to fine size. Observed with polarized light the particles exhibit pleochroism typical of azurite.<br>**2.** Amorphous and brownish translucent layer<br>**3.** Ground made with very coarse to fine aggregates of off white pigment with scattered impurities due to the presence of abundant darkened binding medium.	

Table S2.- Strata distribution and chemical composition of layers found in the set of samples analysed using FESEM-EDX.

<table>
<tr><th colspan="3">C-2</th></tr>
<tr>
<td rowspan="2">

Electron image of the cross-section

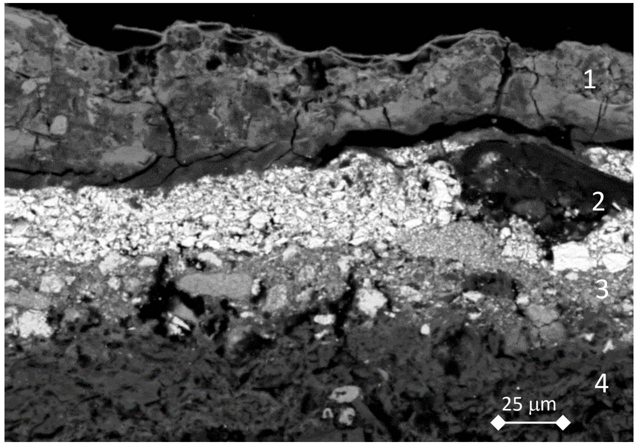

Layer assignment and compounds identified

1. Protective coating: Altered organic compound
2. Paint layer: Cinnabar
3. Ground: Calcium sulfate (gypsum)

</td>
<td>

Layer 1: X-ray spectrum

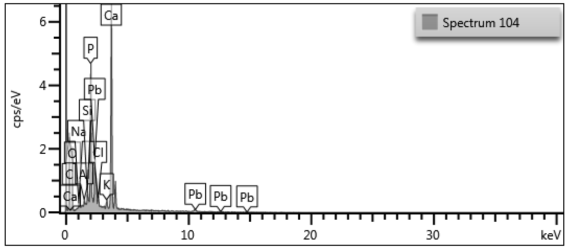

</td>
<td>

Layer 3: X-ray spectrum

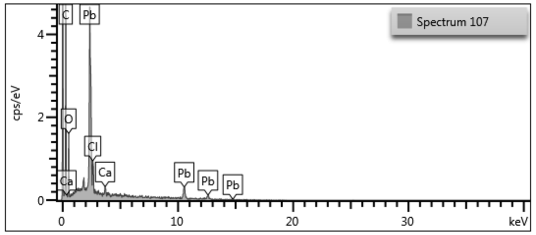

</td>
</tr>
<tr>
<td>

Layer 2: X-ray spectra

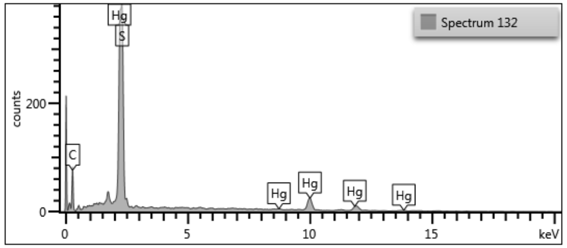

</td>
<td>

Layer 4: X-ray spectrum

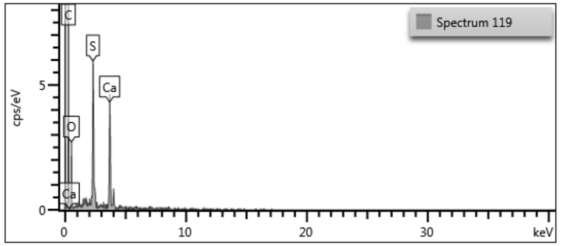

</td>
</tr>
</table>

<table>
<tr><td colspan="3"></td></tr>
<tr><td rowspan="2">Electron image of the cross-section
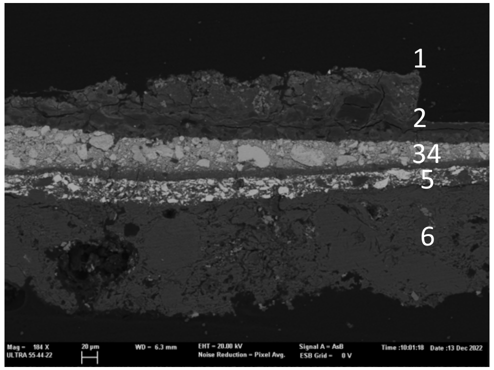
</td><td>Surface: X-ray spectrum
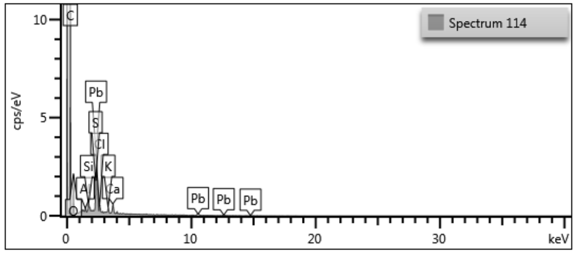
</td><td>Layer 5: X-ray spectrum
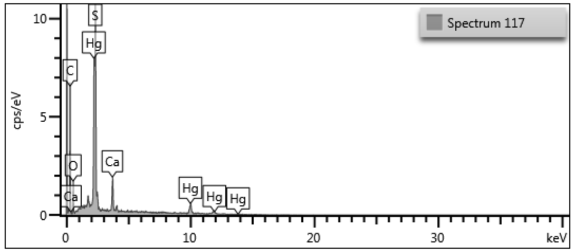
</td></tr>
<tr><td>Layer 2: X-ray spectrum
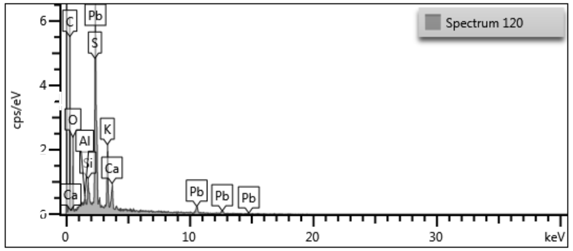
</td><td>Layer 6: X-ray spectrum
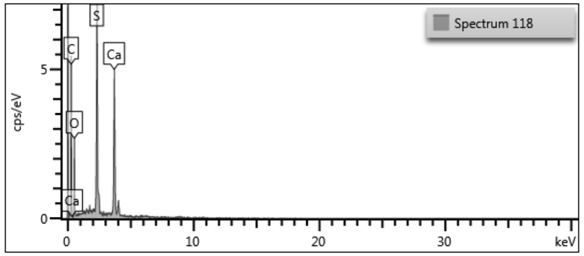
</td></tr>
</table>

**Layer assignment and compounds identified**<br>**1.** Surface: clayey minerals, gypsum, Pb-rich migrated species<br>**2.** Lower coating: organic compounds with Pb-rich migrated species<br>**3.** White paint layer: lead white<br>**4.** Thin organic layer: organic compound<br>**5.** Red paint layer: cinnabar<br>**6.**White ground : calcium sulfate (gypsum)	**Layer 3: X-ray spectrum**<br>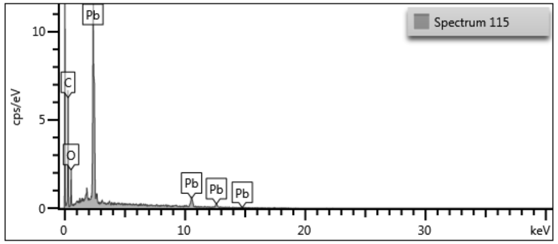	

C-5

Electron image of the cross-section

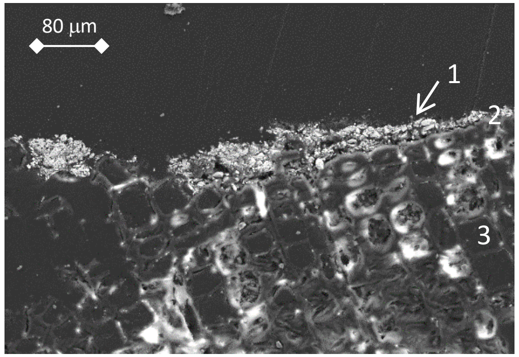

Layer assignment and compounds identified

1. Top paint layer: orpiment and white lead
2. Bottom paint layer: cinnabar
3. Support: wood with cell holes filled with gypsum

Layer 1: X-ray spectrum

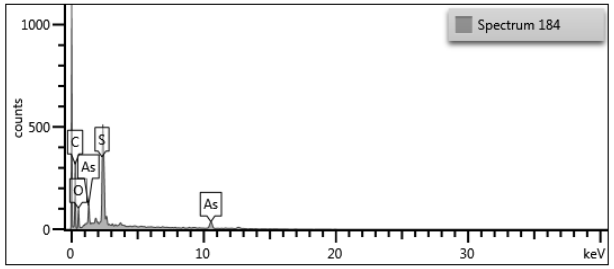

Layer 2: X-ray spectrum

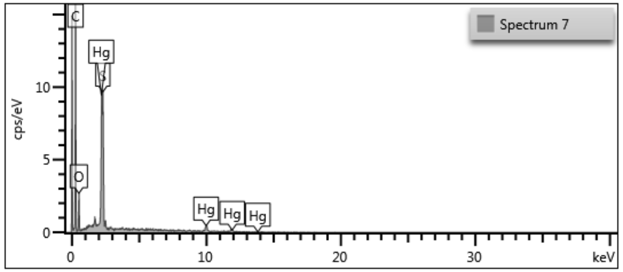

Support 3: X-ray spectrum

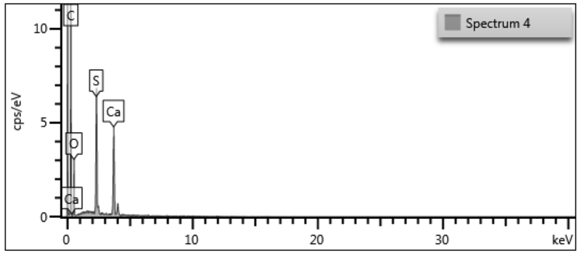

D-1

Electron image of the cross-section

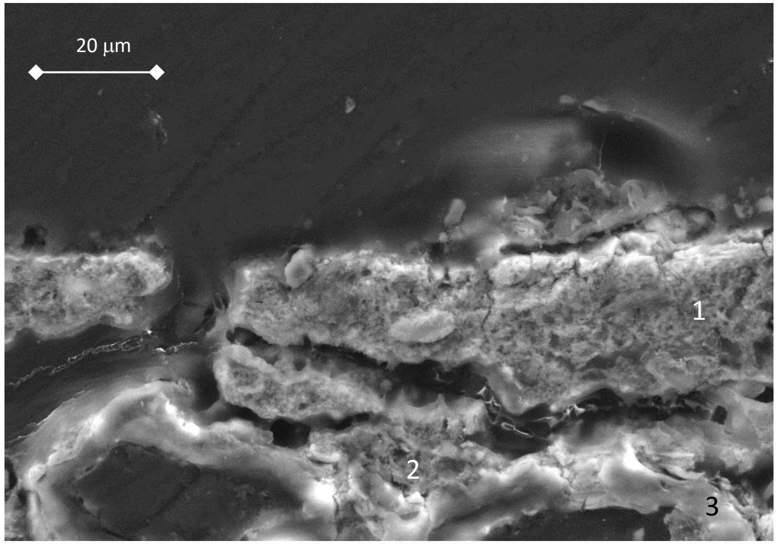

Layer assignment and compounds identified

1. Top paint layer: orpiment
2. Bottom paint layer: indigo
3. Support: wood

Layer 1: X-ray spectrum

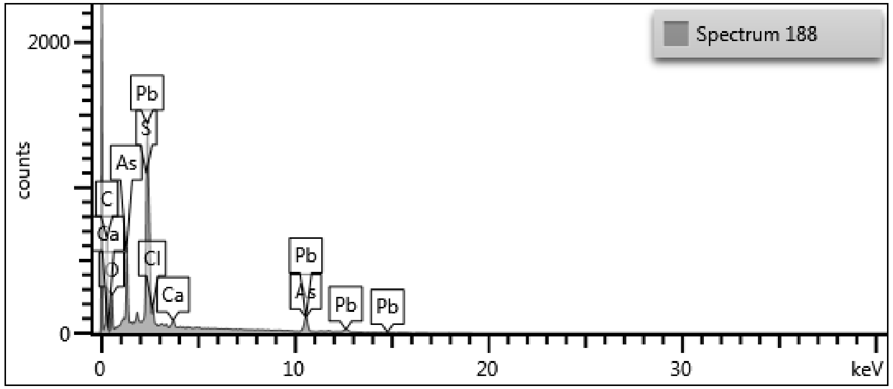

<table>
<tr><th colspan="3">F-1</th></tr>
<tr>
<td rowspan="2">Electron image of the cross-section
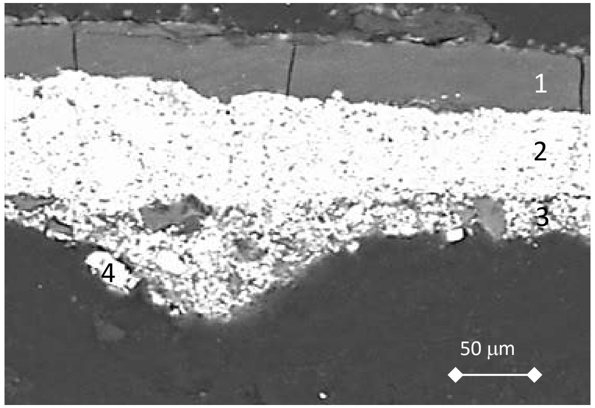

Layer assignment and compounds identified
1. Protective coating: organic compound
2. Top paint layer: lead white
3. Middle paint layer: lead white, indigo
4. Bottom paint layer: cinnabar</td>
<td>Layer 2: X-ray spectrum
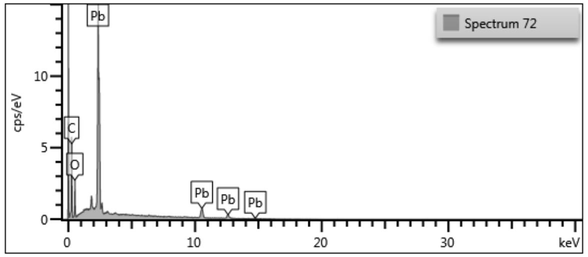
</td>
<td>Layer 4: X-ray spectrum
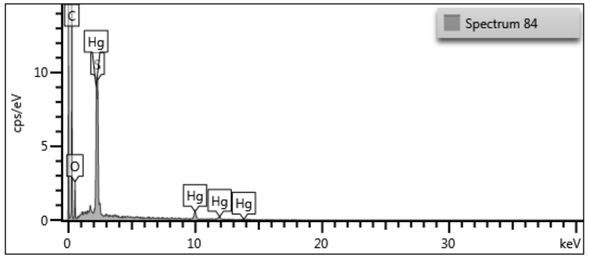
</td>
</tr>
<tr>
<td>Layer 3: X-ray spectrum
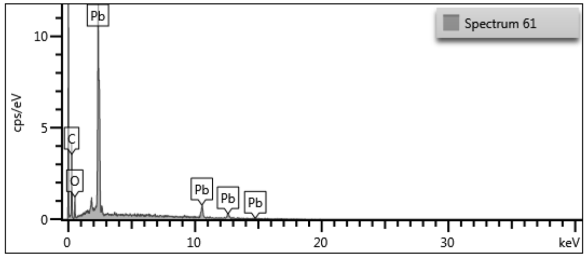
</td>
<td></td>
</tr>
</table>

<table>
<tr><th colspan="3">F-4</th></tr>
<tr><td>Electron image of the cross-section
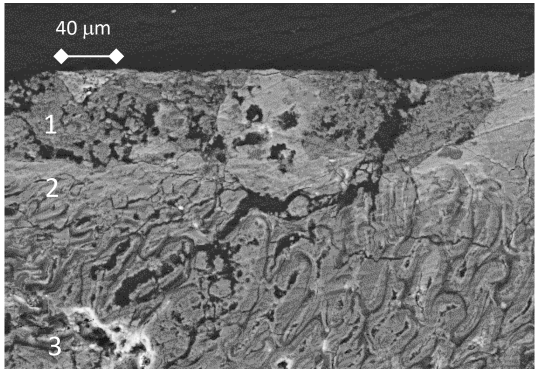

Layer assignment and compounds identified
1. Top paint layer: lead white, organic blue pigment
2. Ground: wood support</td><td>Layer 1: X-ray spectrum
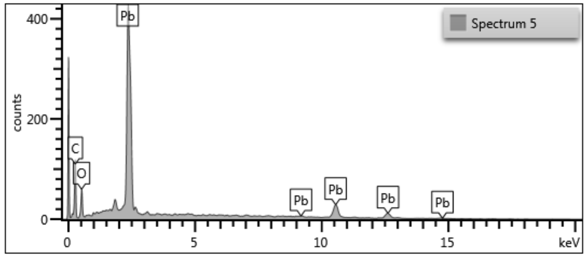
</td><td>Layer 2: X-ray spectrum</td></tr>
</table>

J-2

Electron image of the cross-section

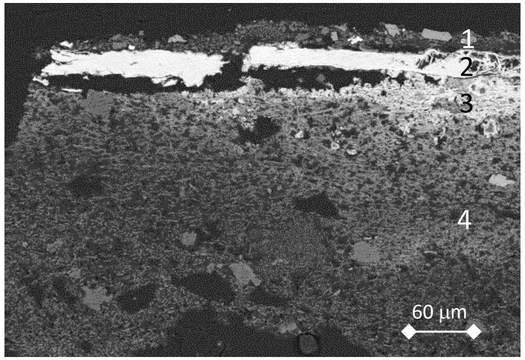

Layer assignment and compounds identified

1. Top paint layer: azurite

2. Metal foil :tin 2

3. Altered metal foil

4. Ground: calcium sulfate (gypsum) 3

100 µm

Layer 1: X-ray spectra

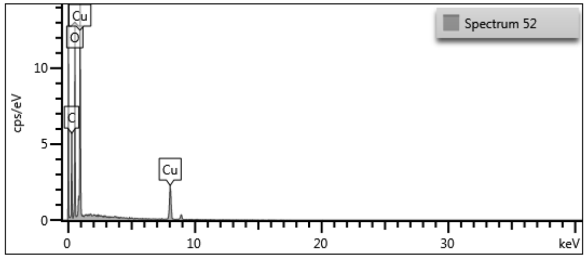

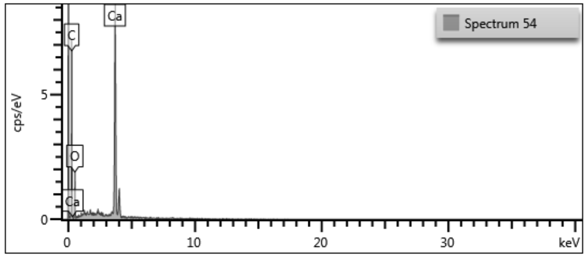

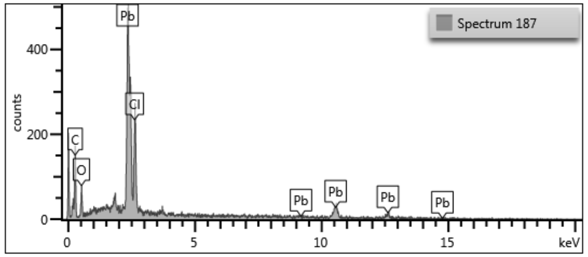

Layer 2: X-ray spectrum

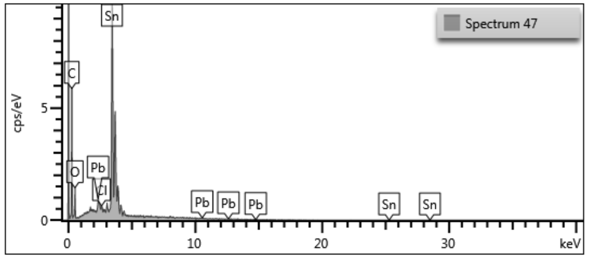

Layer 3: X-ray spectrum

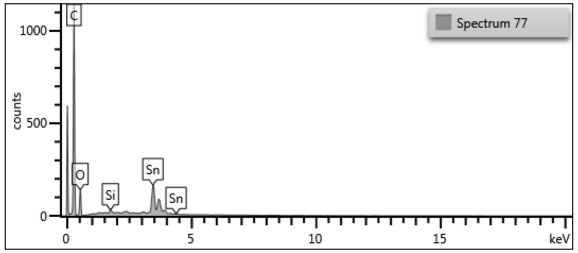

Layer 4: X-ray spectrum

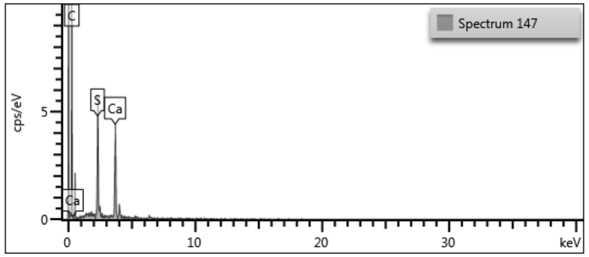

J-3

Electron image of the cross-section

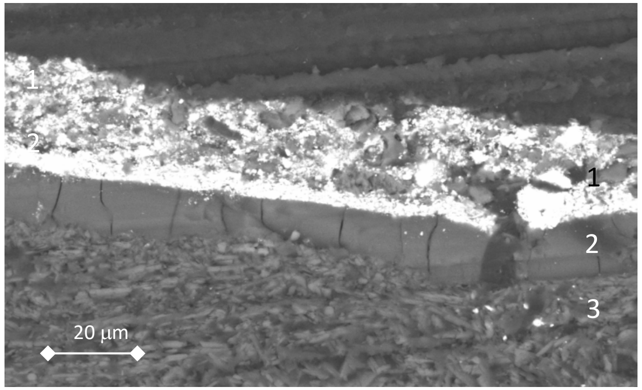

Layer assignment and compounds identified

1. Top paint layer: azurite and lead white
2. Middle paint layer: lead white
3. Bottom paint layer: organic layer
4. Ground: calcium sulfate (gypsum)

Layer 1: X-ray spectra

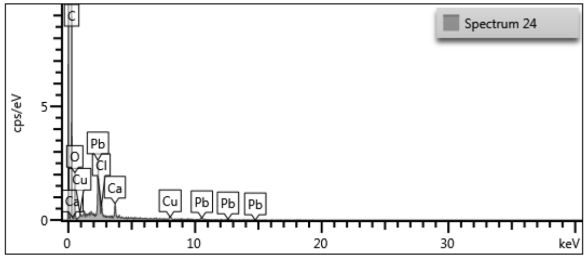

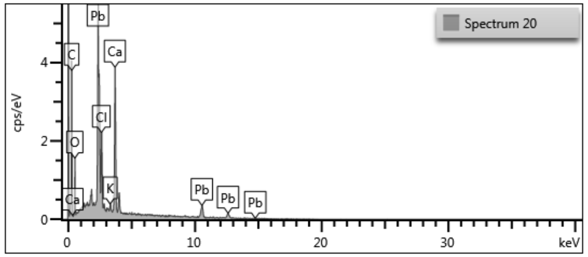

Layer 2: X-ray spectrum

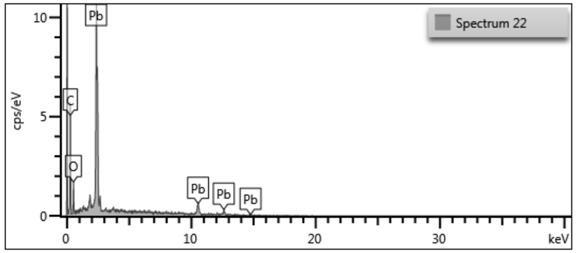

Layer 4: X-ray spectrum

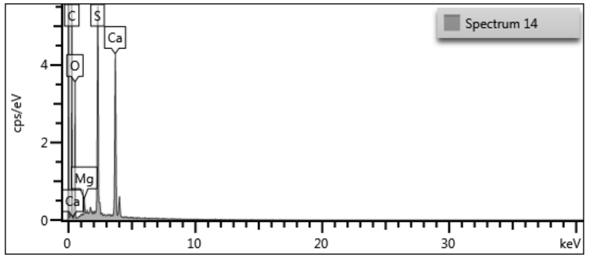

J-4

Electron image of the cross-section

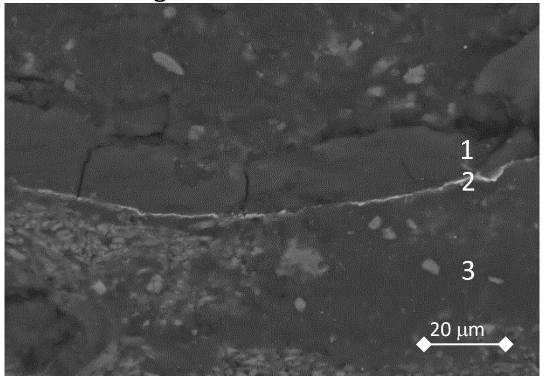

Layer assignment and compounds identified

1. Mineral deposits: clayey materials and calcite
2. Organic coating layer
3. Metal foil: silver altered to silver sulfide
4. Ground: calcium sulfate (gypsum)

Layer 1: X-ray spectra

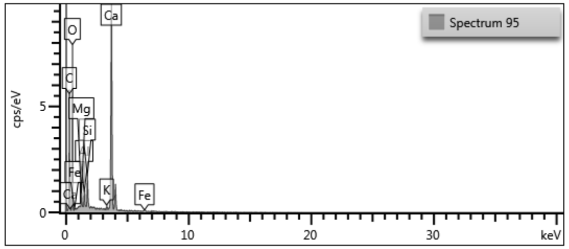

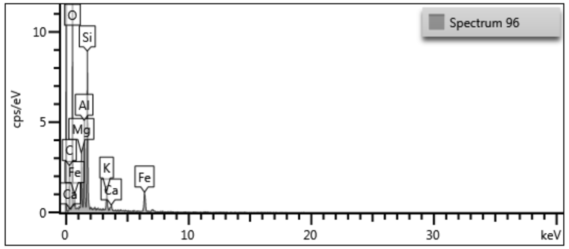

Layer 2: X-ray spectrum

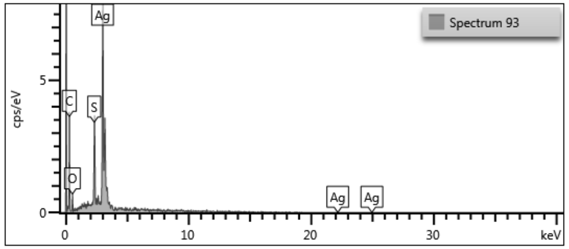

Layer 3: X-ray spectrum

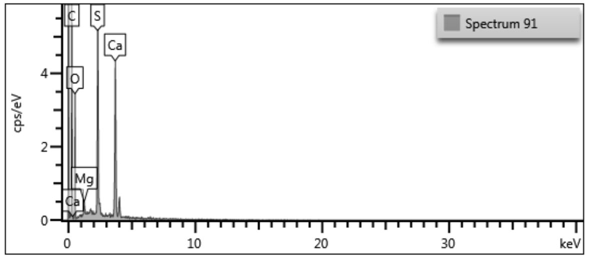

K-4

Electron image of the cross-section

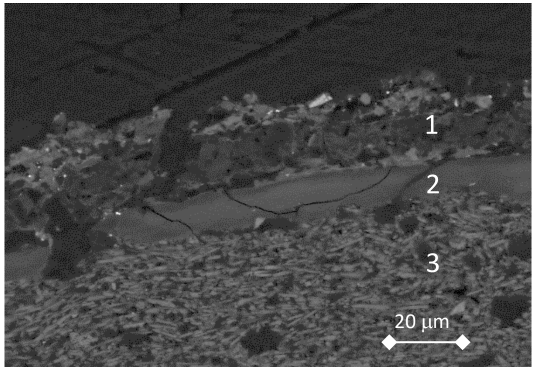

Layer assignment and compounds identified

1. Paint layer: Azurite and lead white
2. Organic layer
3. Ground: calcium sulfate (gypsum with eventual grains of strontium sulfate)

Layer 1: X-ray spectrum

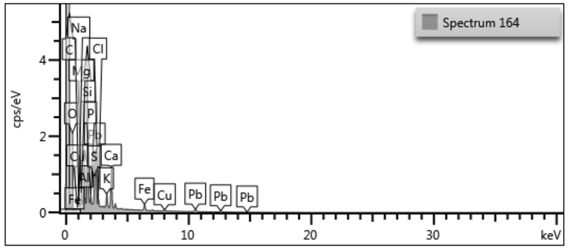

Layer 3: X-ray spectra

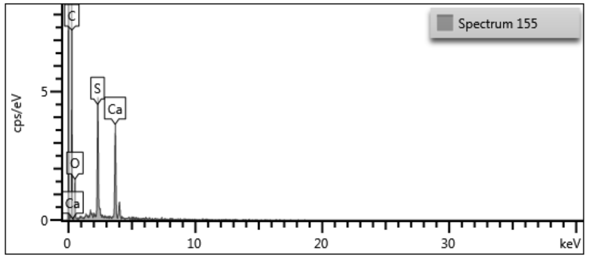

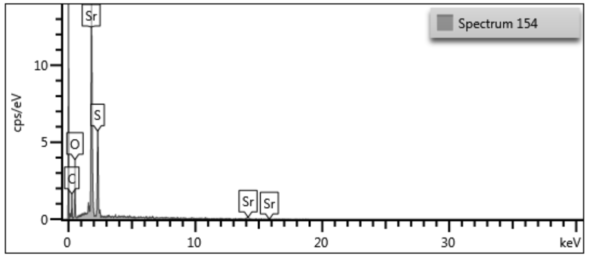

Figure S1.- IR spectrum obtained in the paint layer of sample C-2.

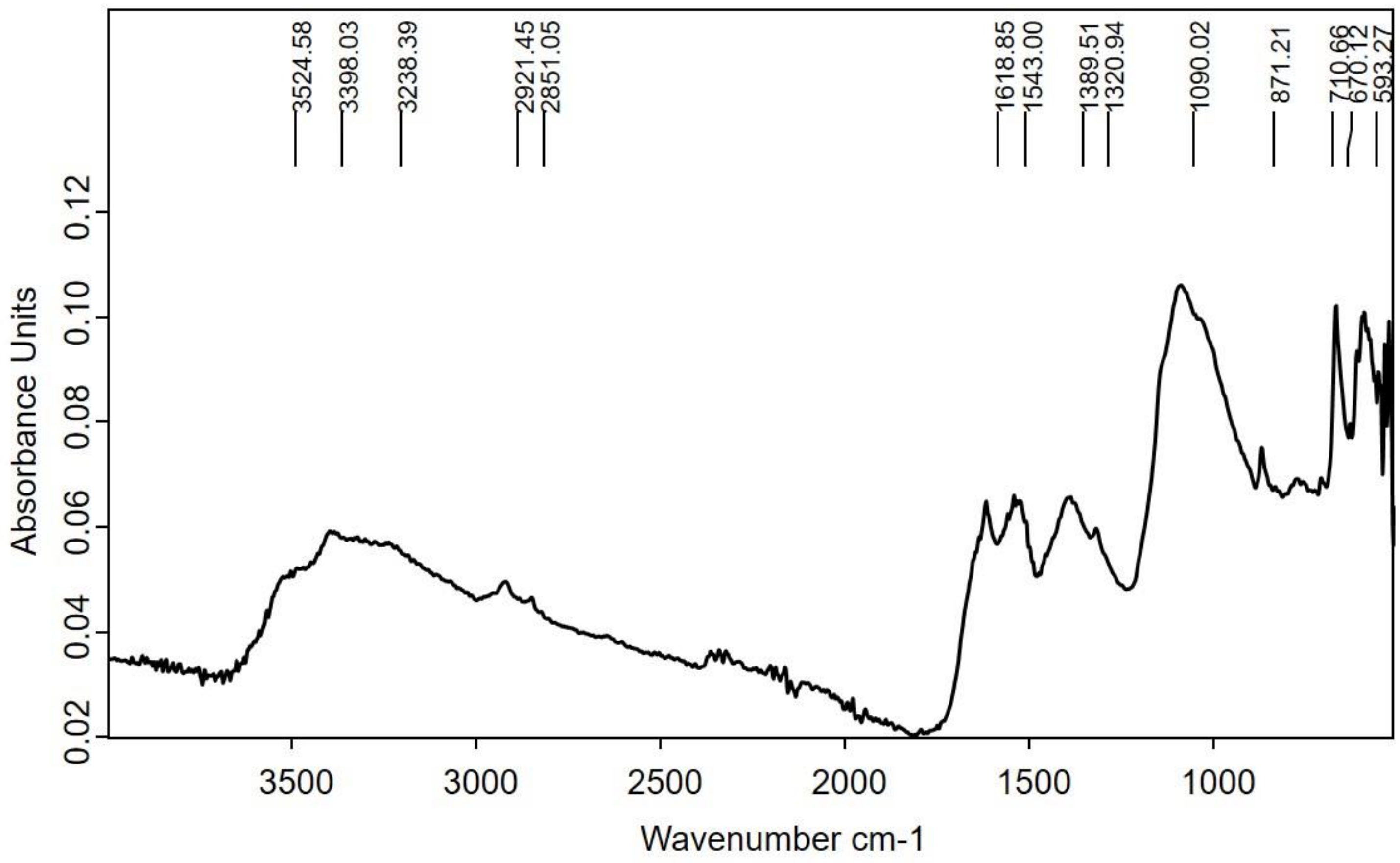

Figure S2.- Detail of the previous IR spectrum obtained in the paint layer of sample C-2.

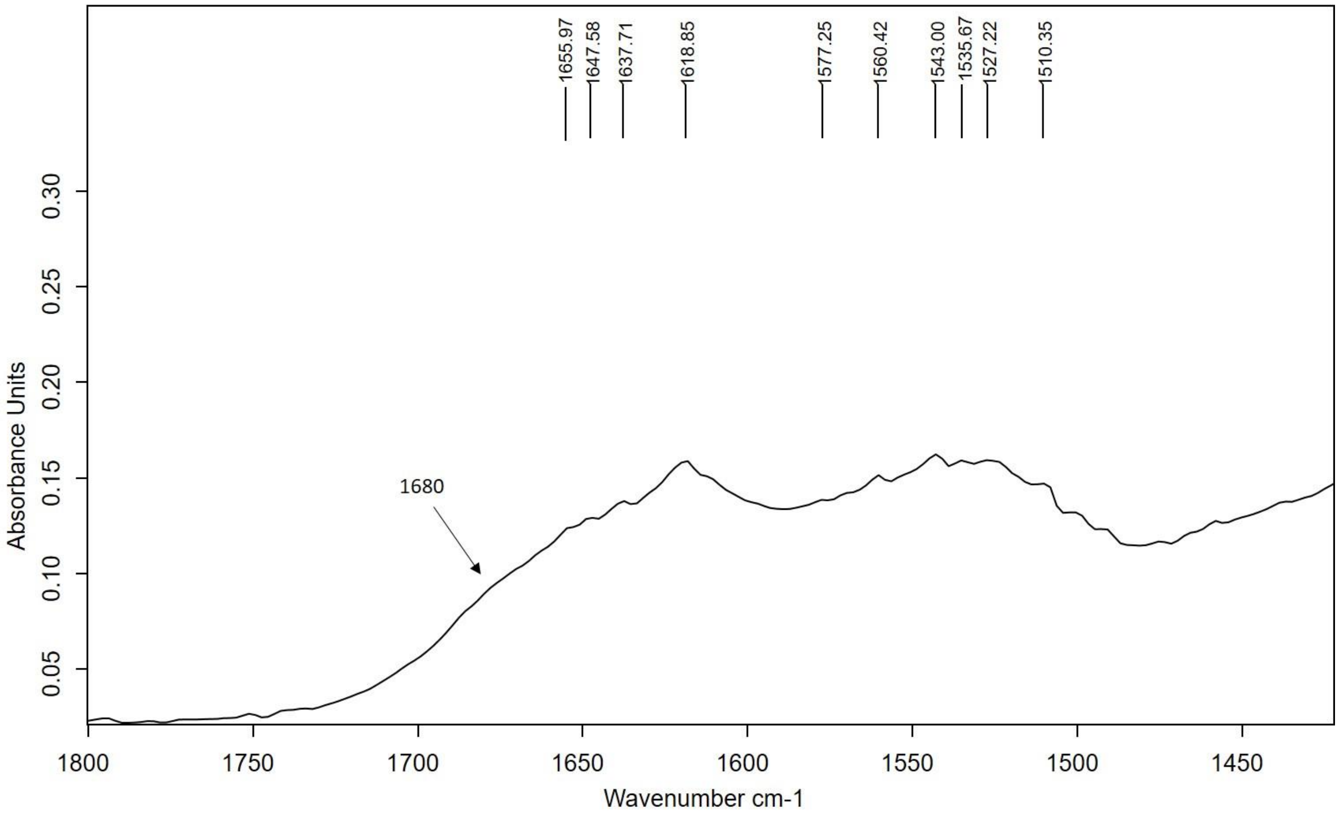

Figure S3.- IR spectrum obtained in the ground of sample C-2.

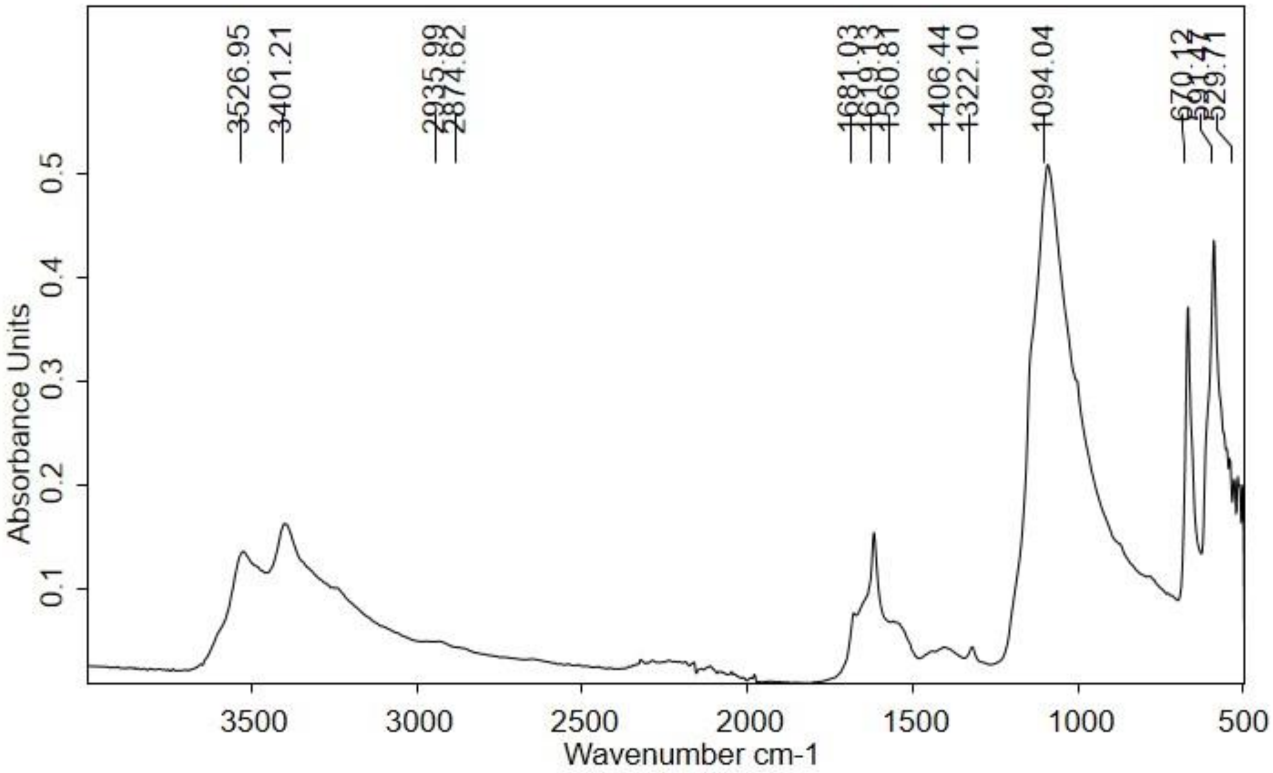

Figure S4.- IR spectrum obtained in the upper paint layer of sample C-4.

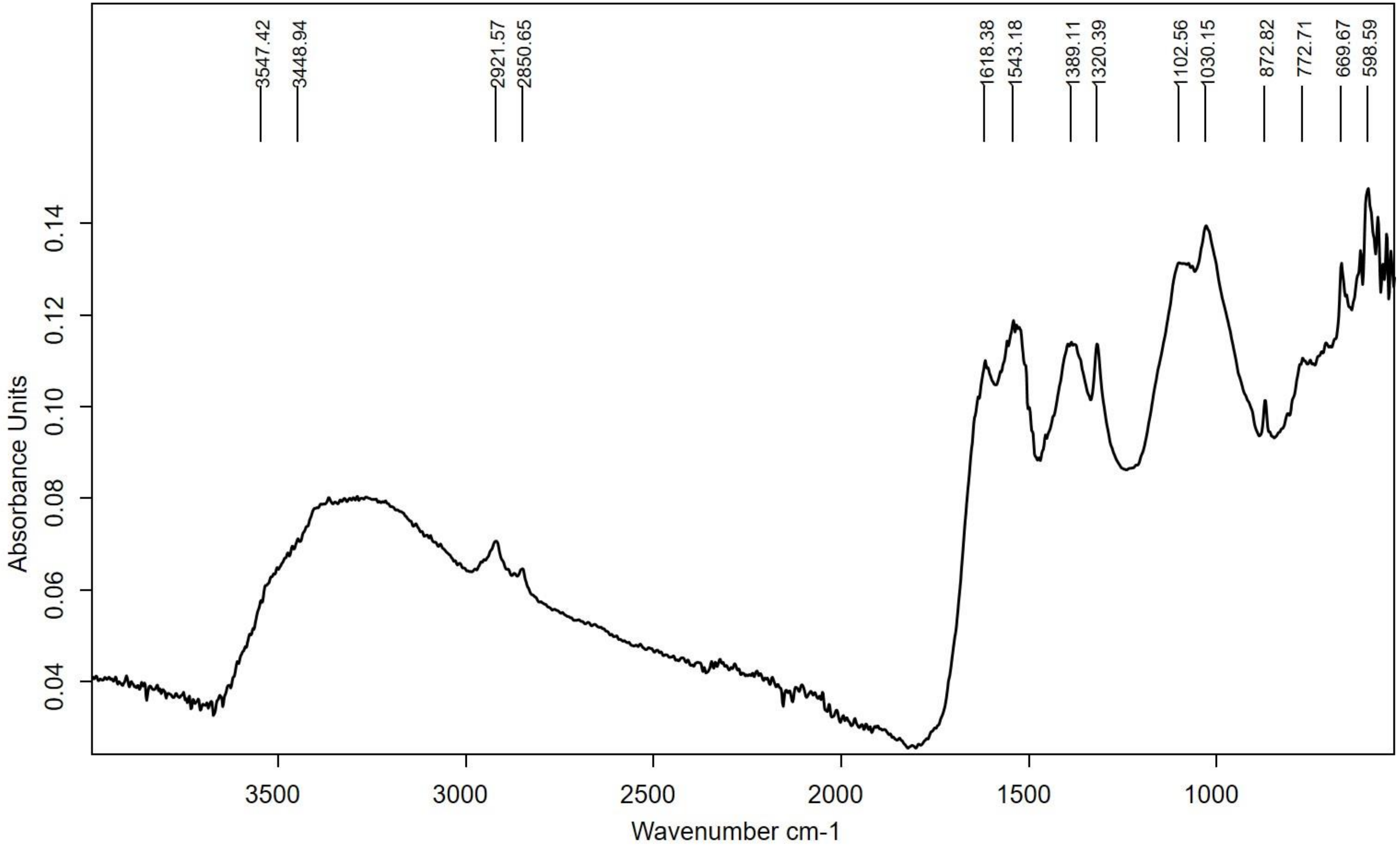

Figure S5.- Detail of the previous IR spectrum obtained in the stretch methyl, methylene region of the paint layer in sample C-4.

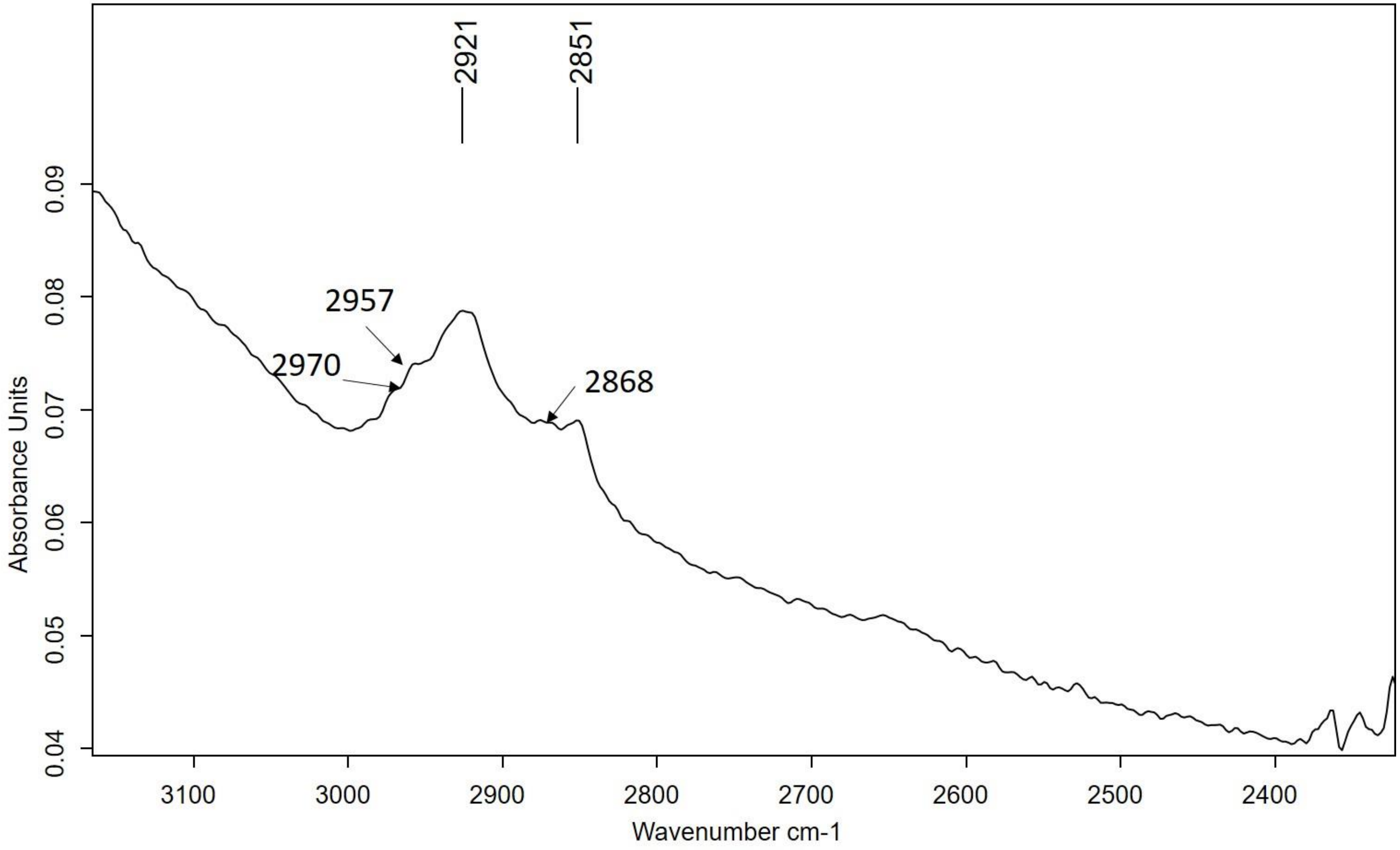

Figure S6.- Detail of the previous IR spectrum obtained in the amide I region of the paint layer in sample C-4.

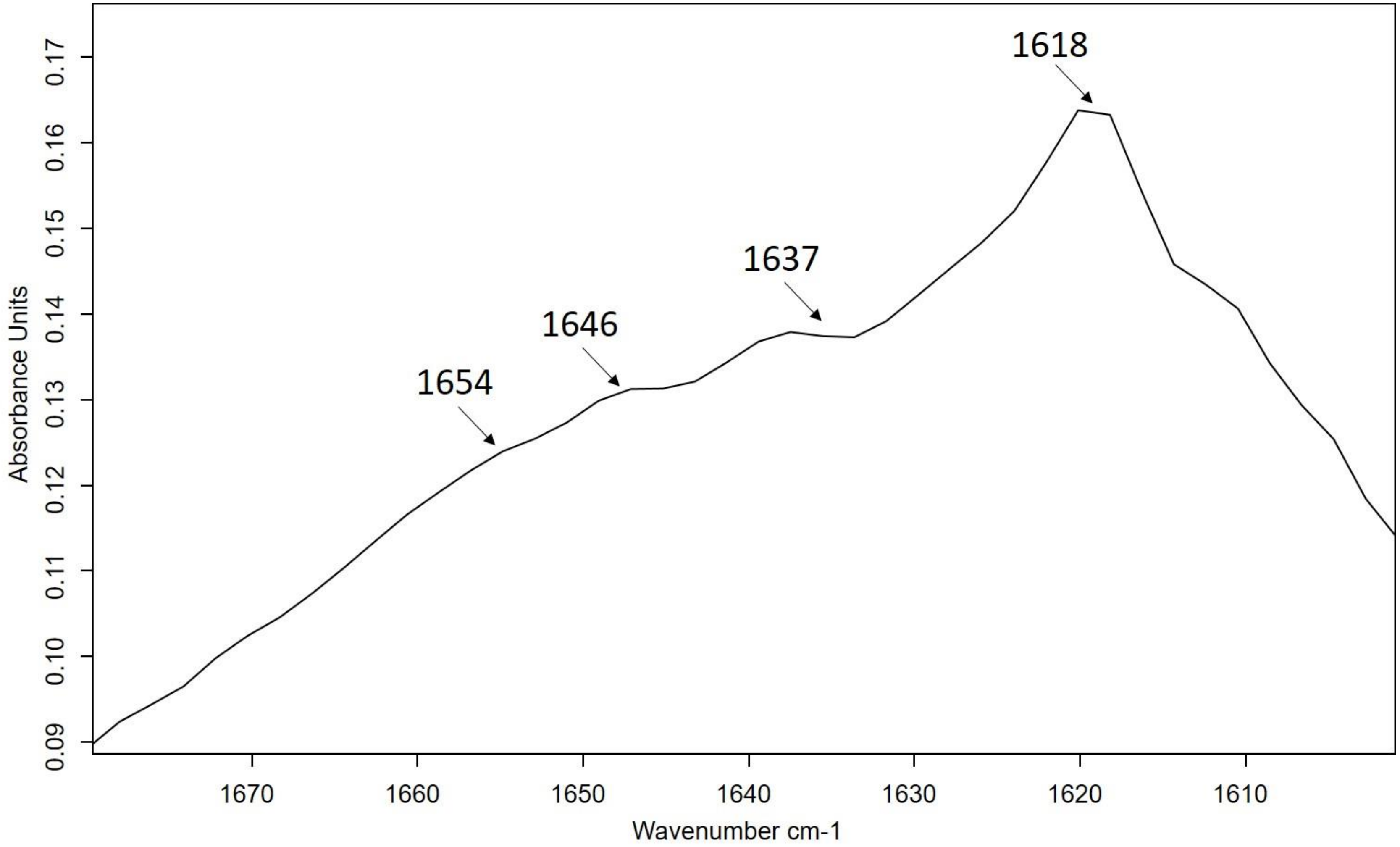

Figure S7.- IR spectrum obtained in the ground of sample C-4.

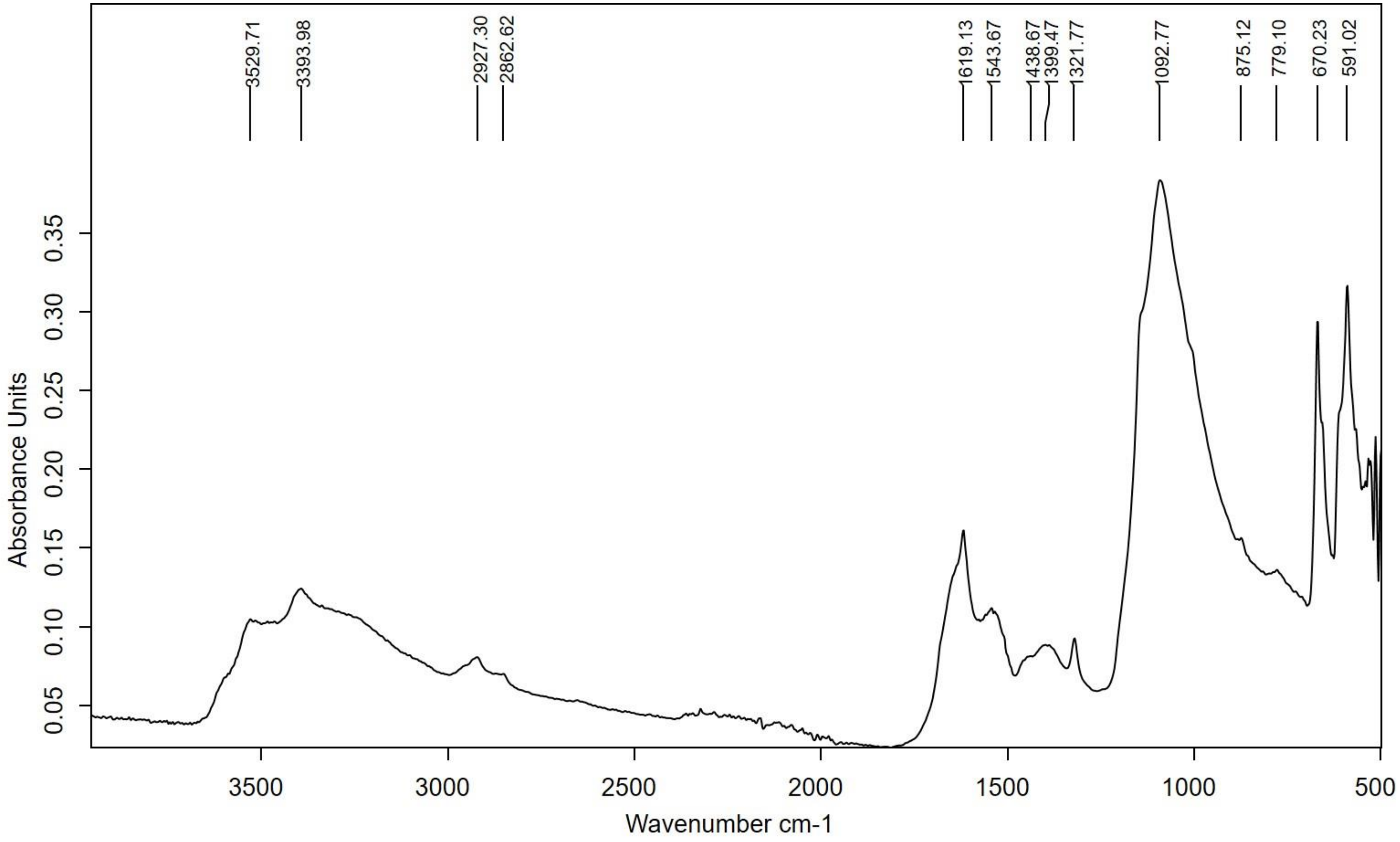

Figure S8.- IR spectrum obtained in the paint layer of sample C-5.

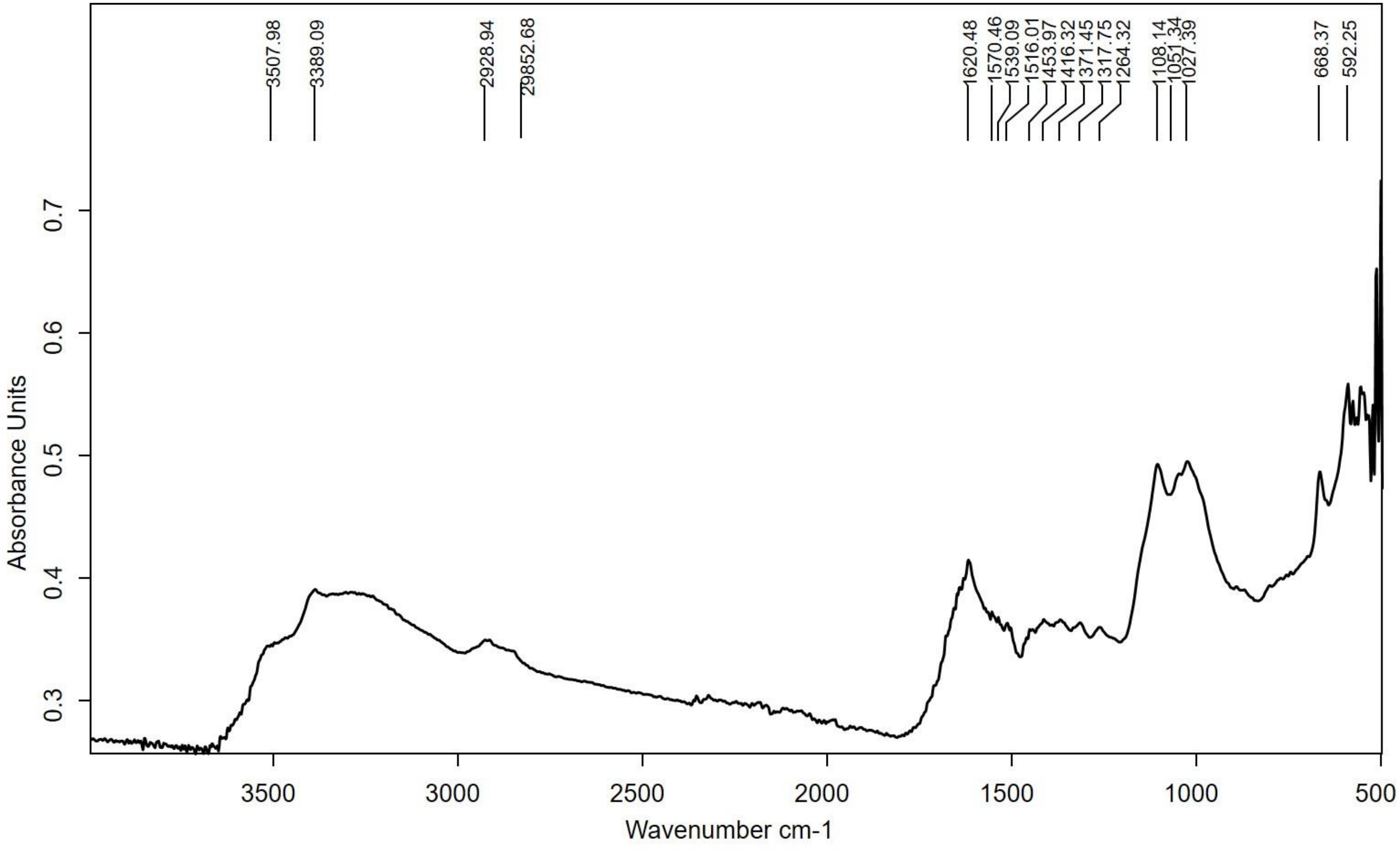

Figure S9.- Detail of the previous IR spectrum obtained in the amide I region of the paint layer in sample C-5.

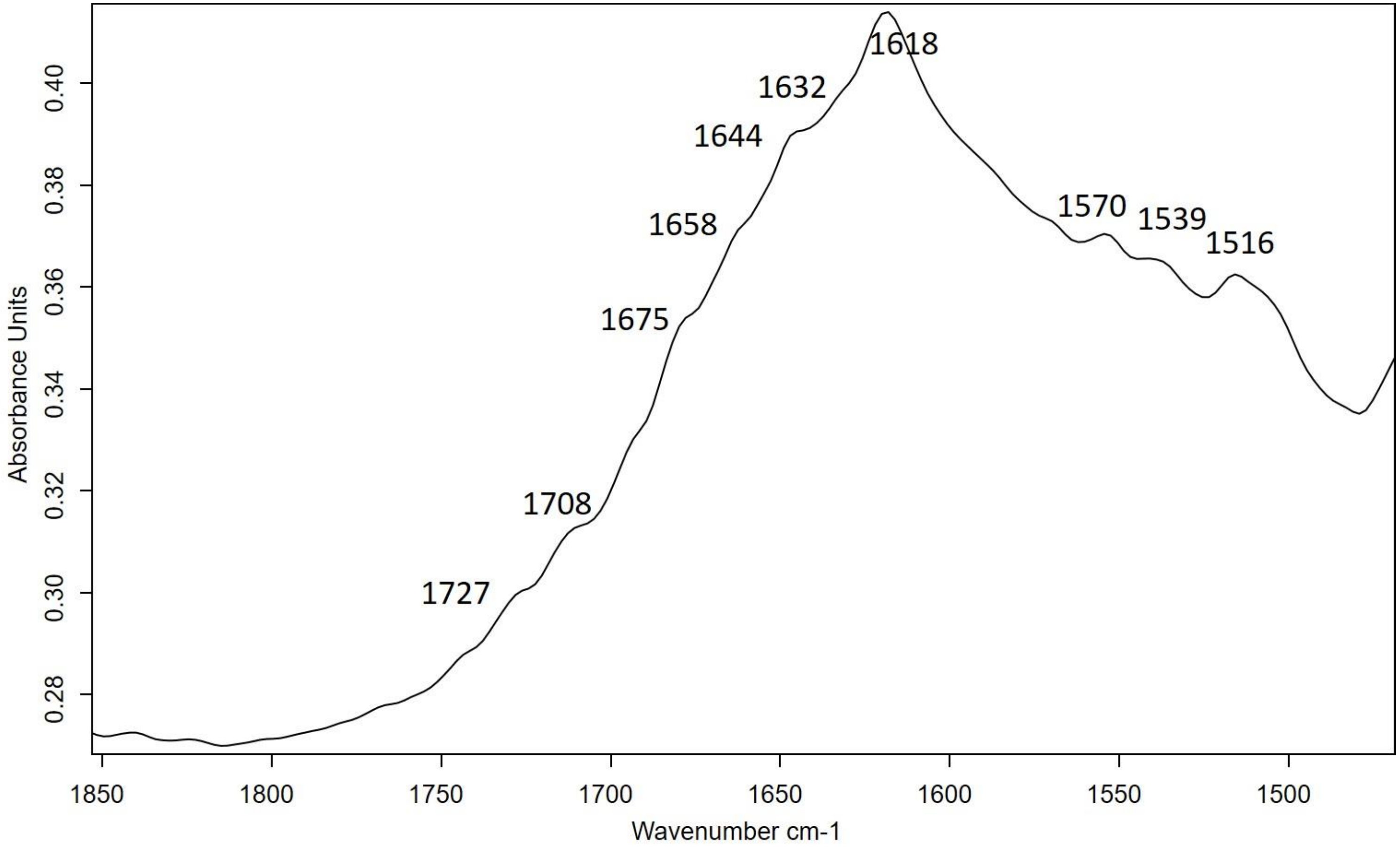

Figure S10.- IR spectrum obtained in the paint layer of sample D-1.

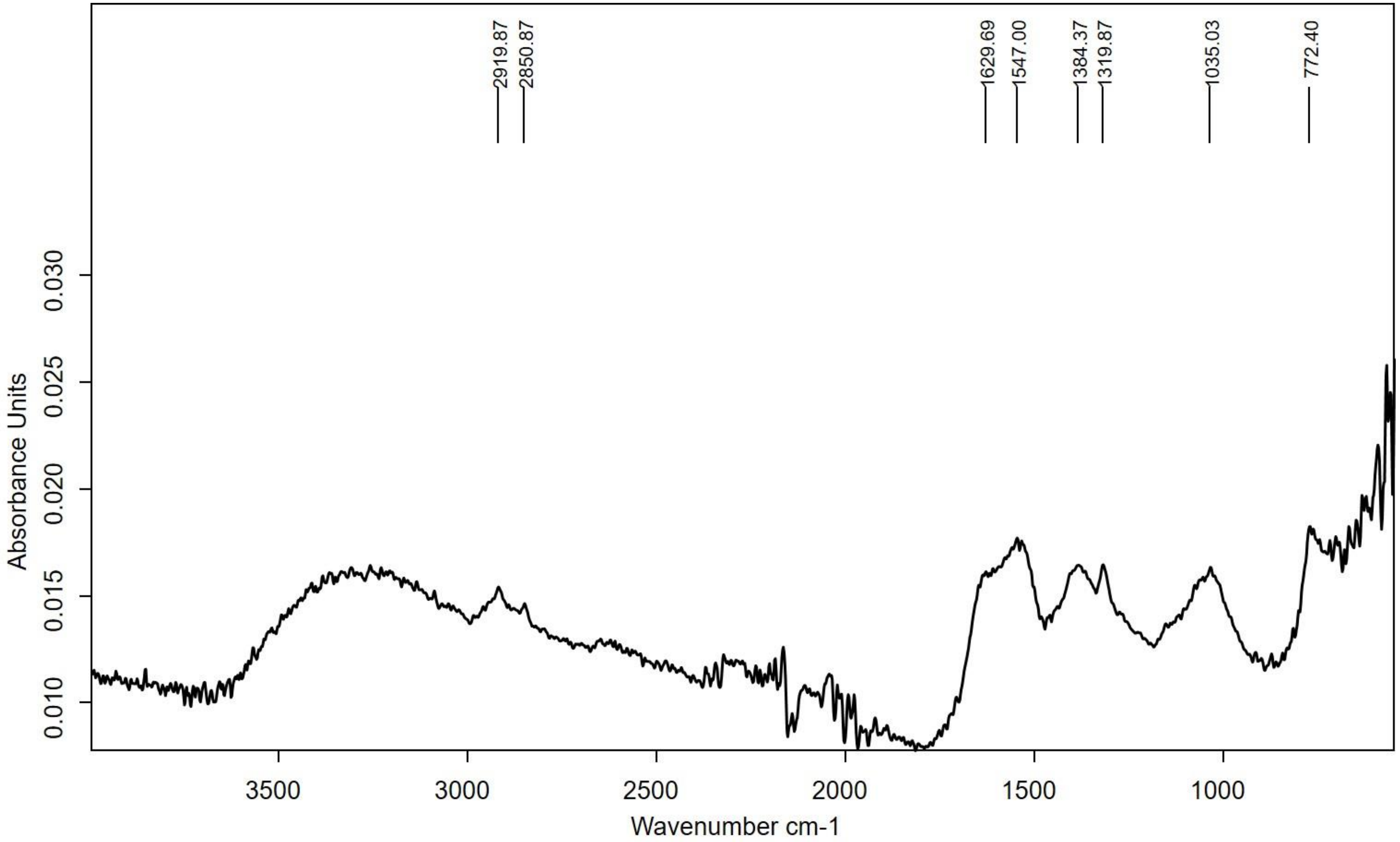

Figure S11.- Detail of the previous IR spectrum obtained in the amide I region of the paint layer in sample D-1.

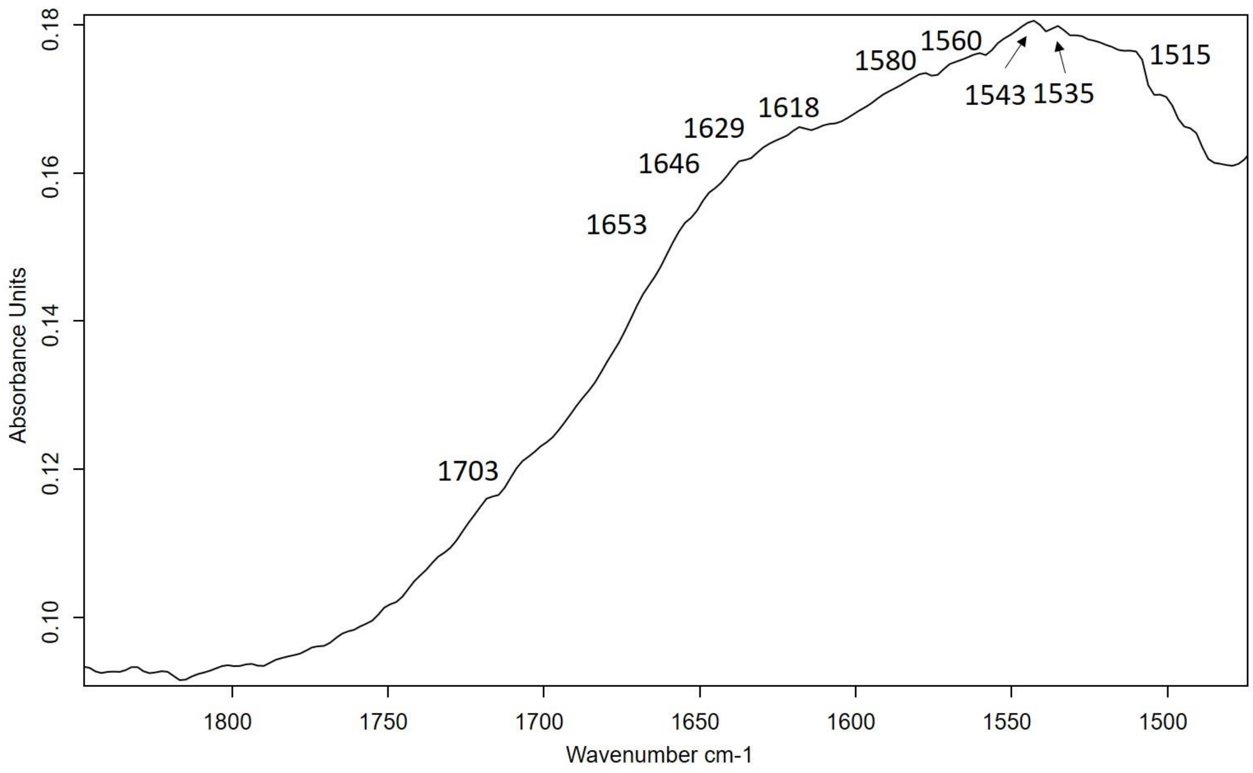

Figure S12.- IR spectrum obtained in the paint layers of sample F-1.

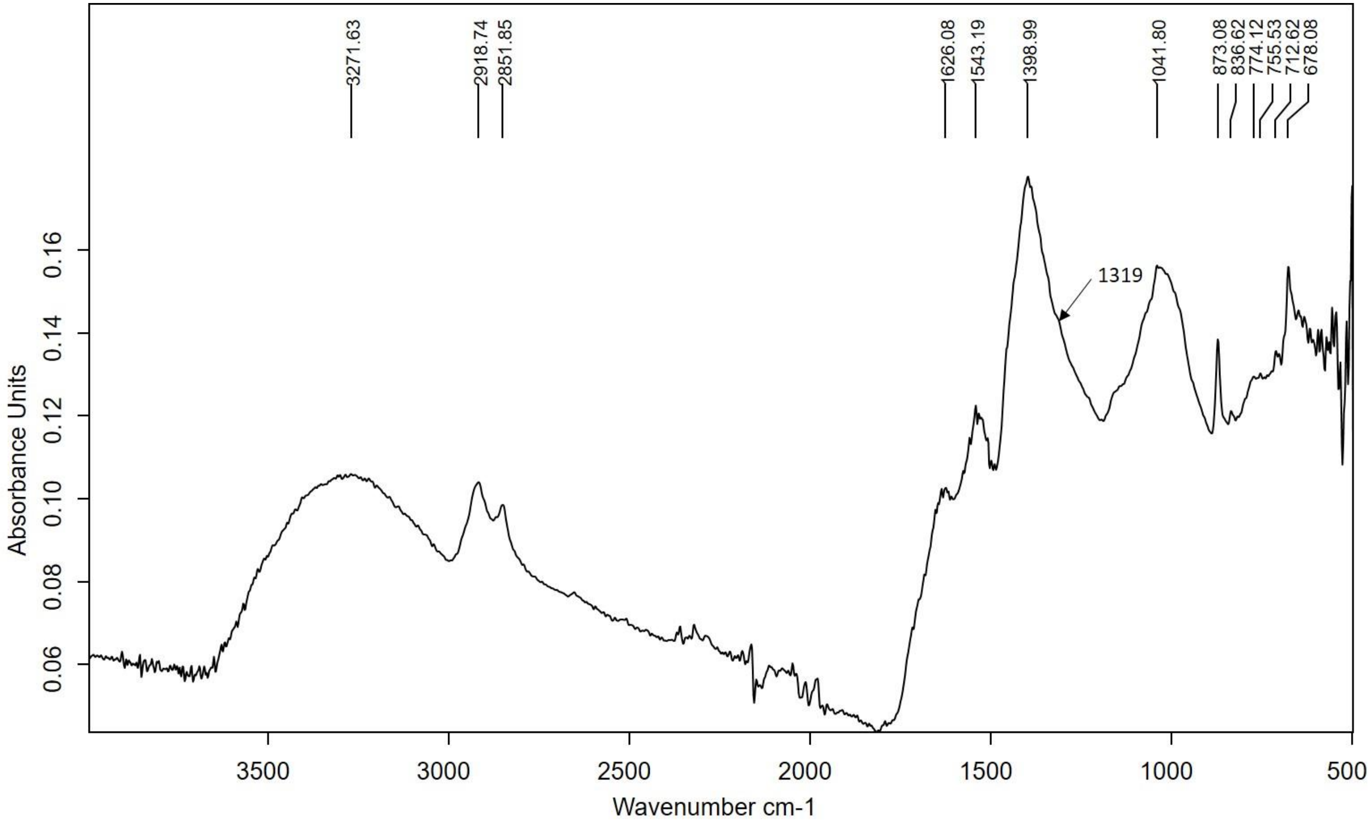

Figure S13.- Detail of the previous IR spectrum obtained in the amide I region of the paint layer in sample F-1.

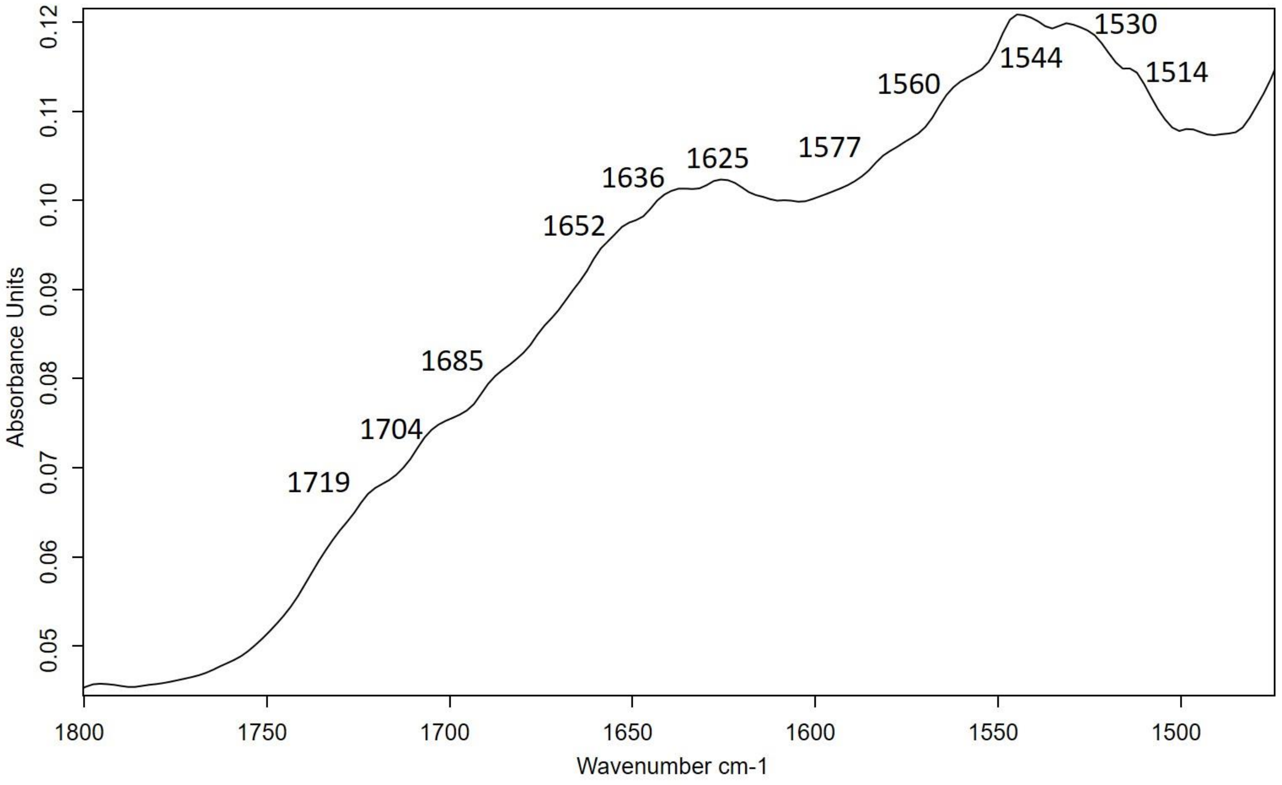

Figure S14.- IR spectrum obtained in the paint layers of sample F-4.

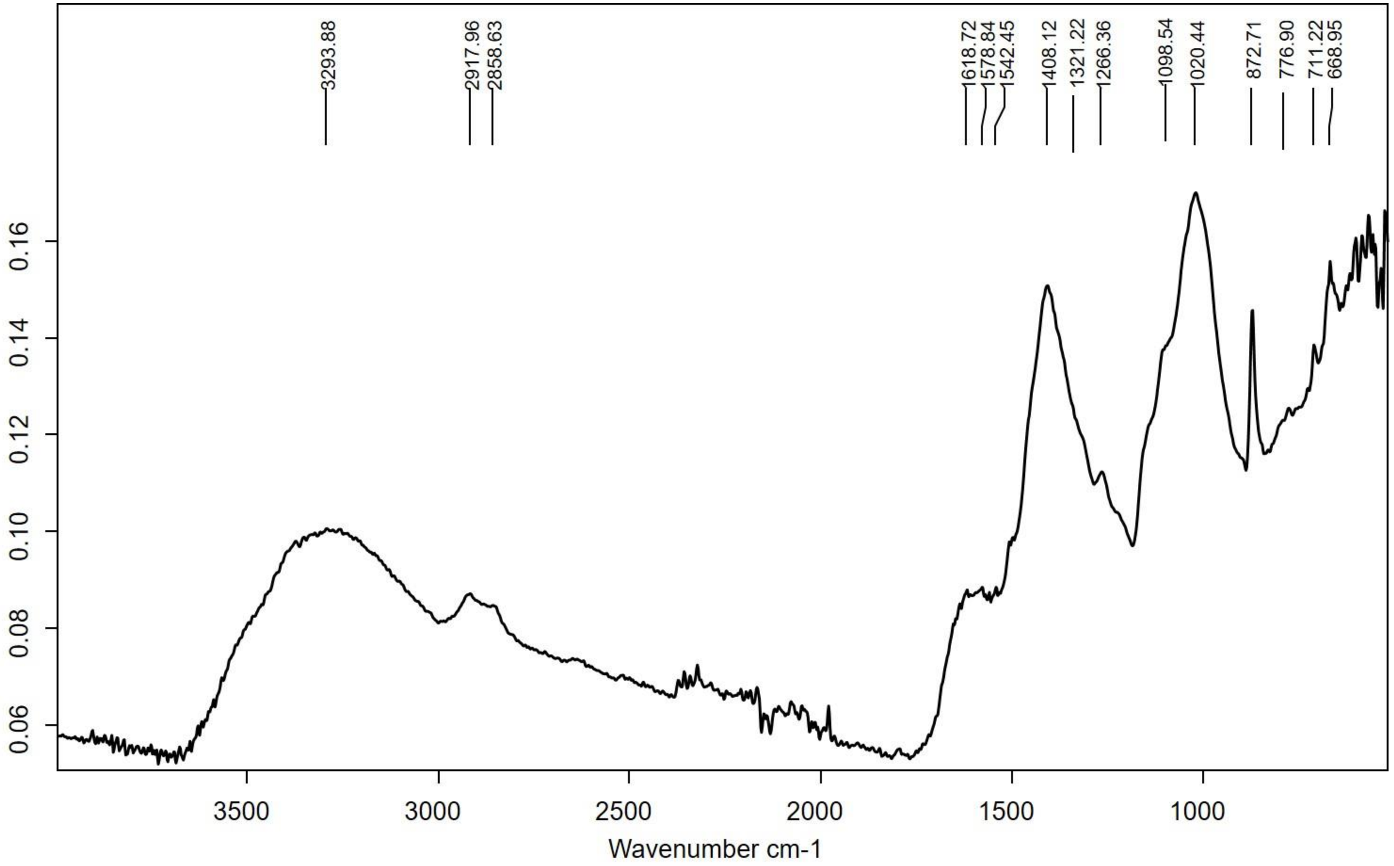

Figure S15.- Detail of the previous IR spectrum obtained in the amide I region of the paint layer in sample F-4.

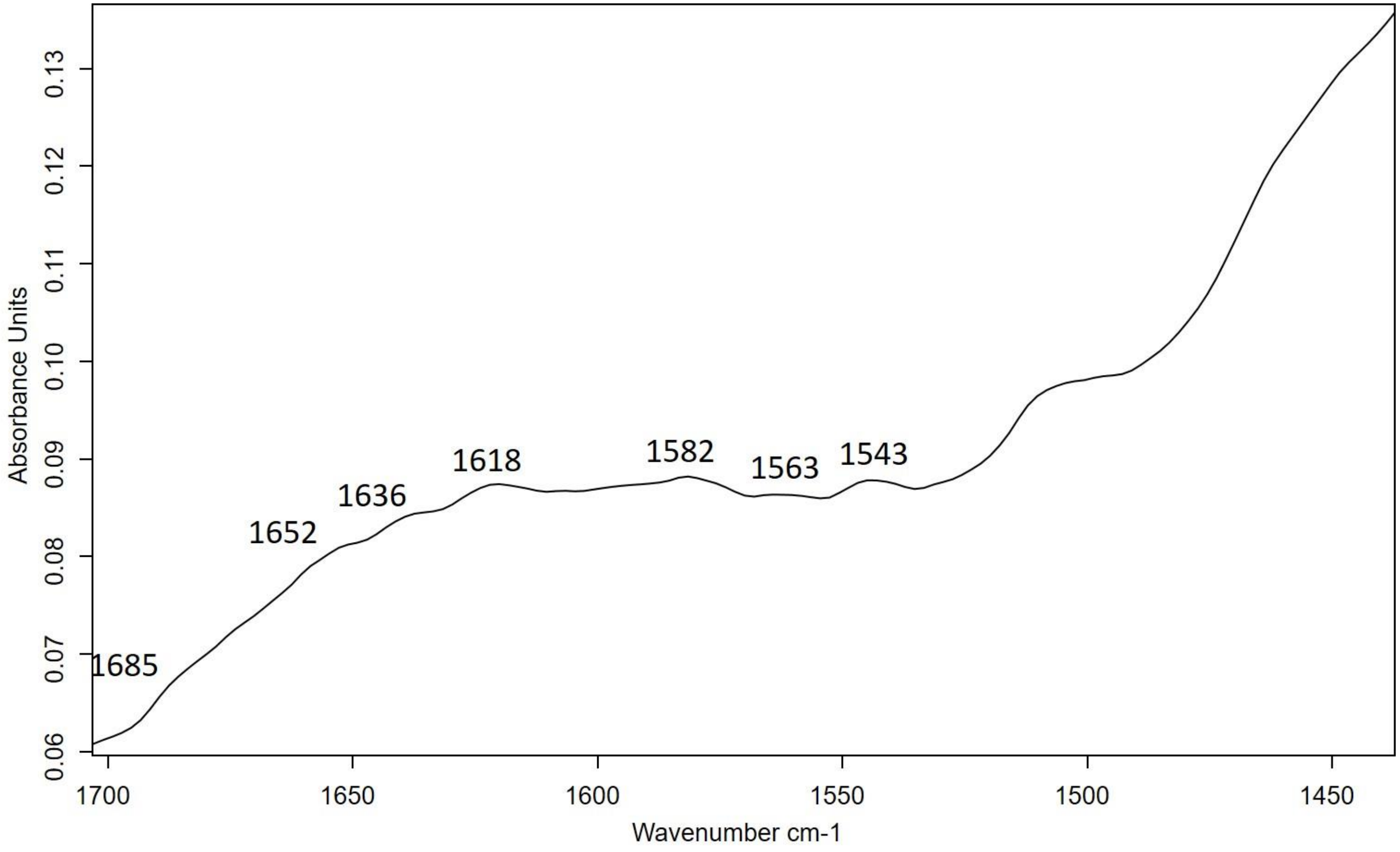

Figure S16.- IR spectrum obtained in the ground of sample F-4.

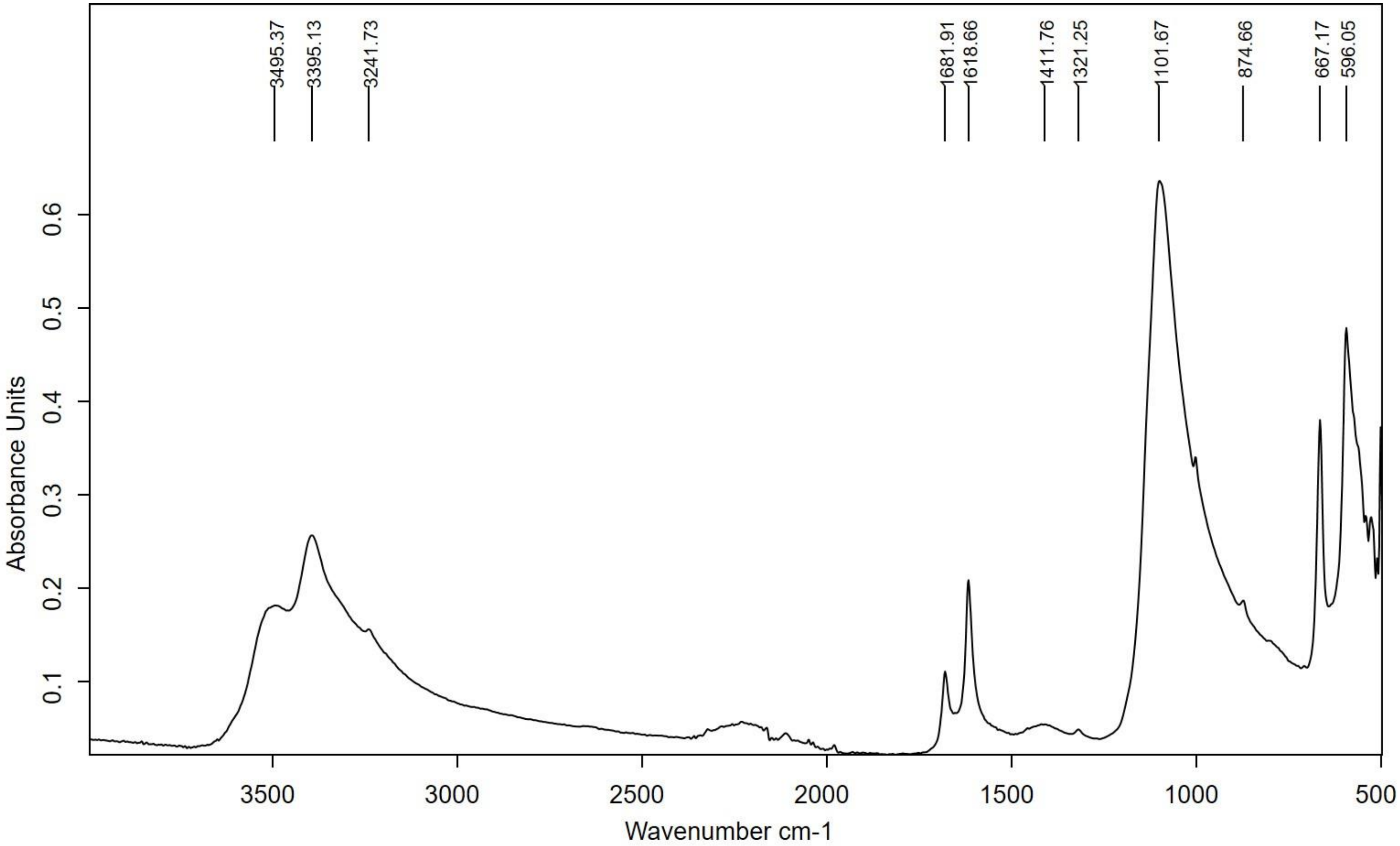

Figure S17.- IR spectrum obtained in the paint layers of sample J-2.

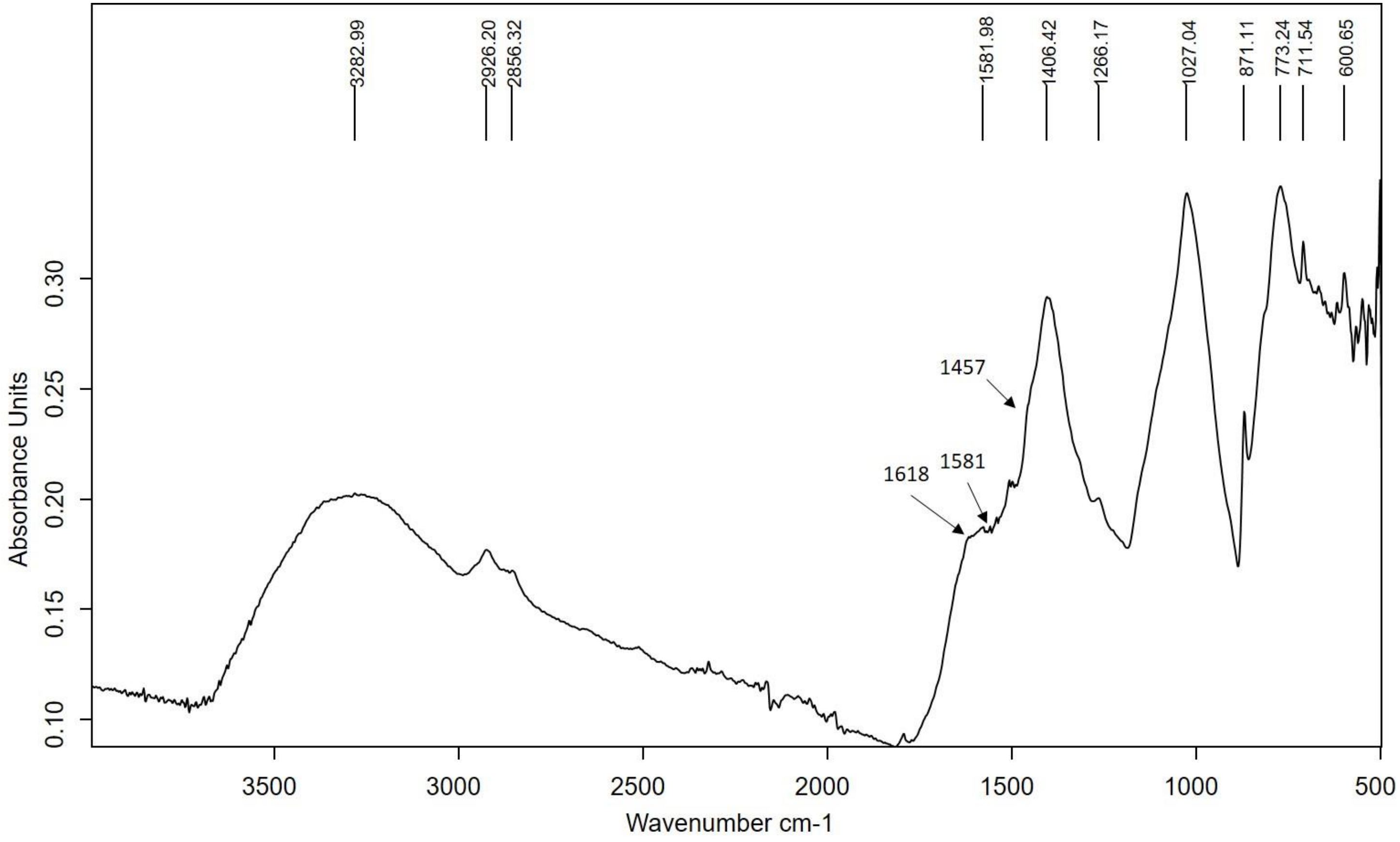

Figure S18.- Detail of the previous IR spectrum obtained in the amide I region of the paint layer in sample J-2.

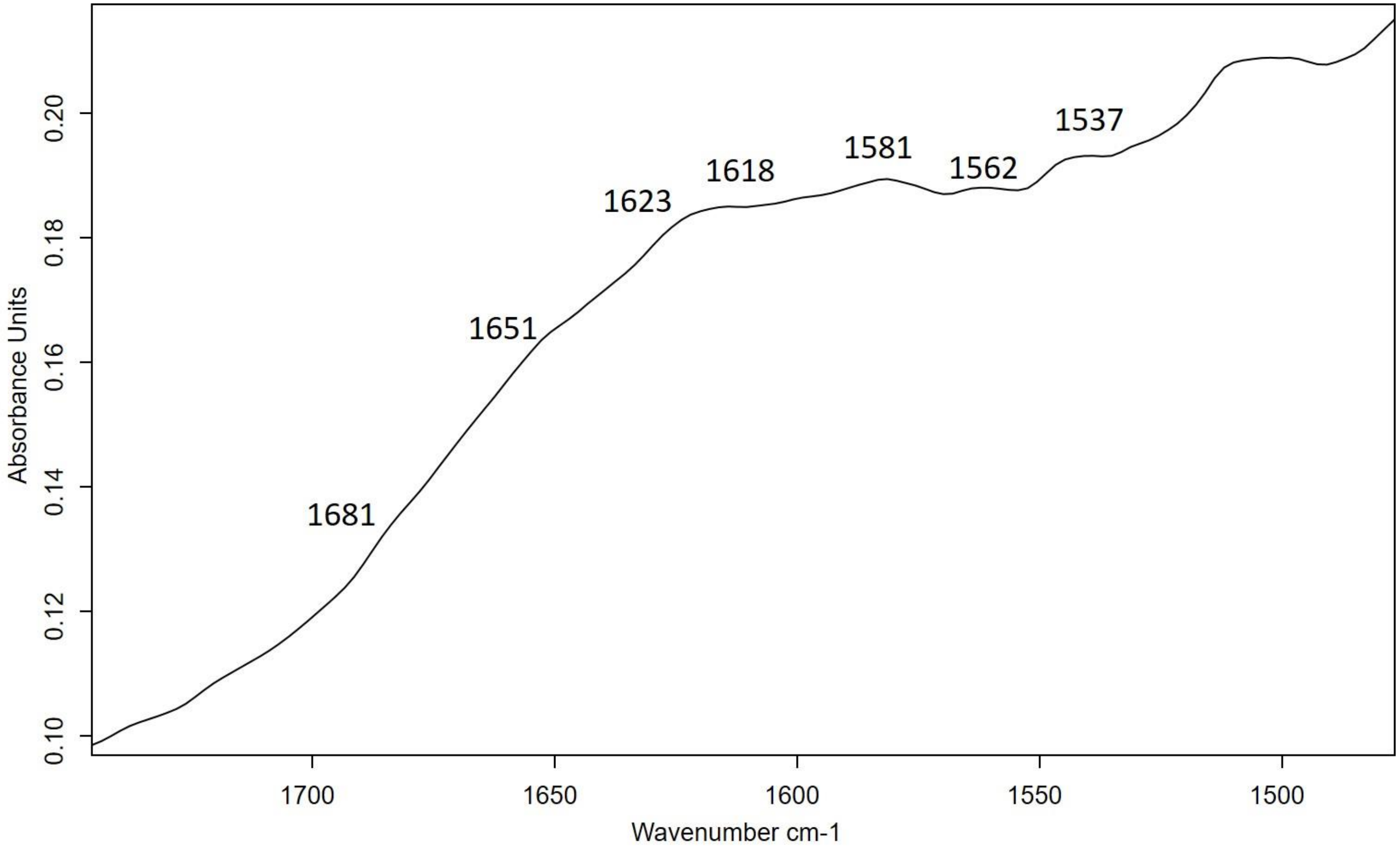

Figure S19.- IR spectrum obtained in the ground of sample J-2.

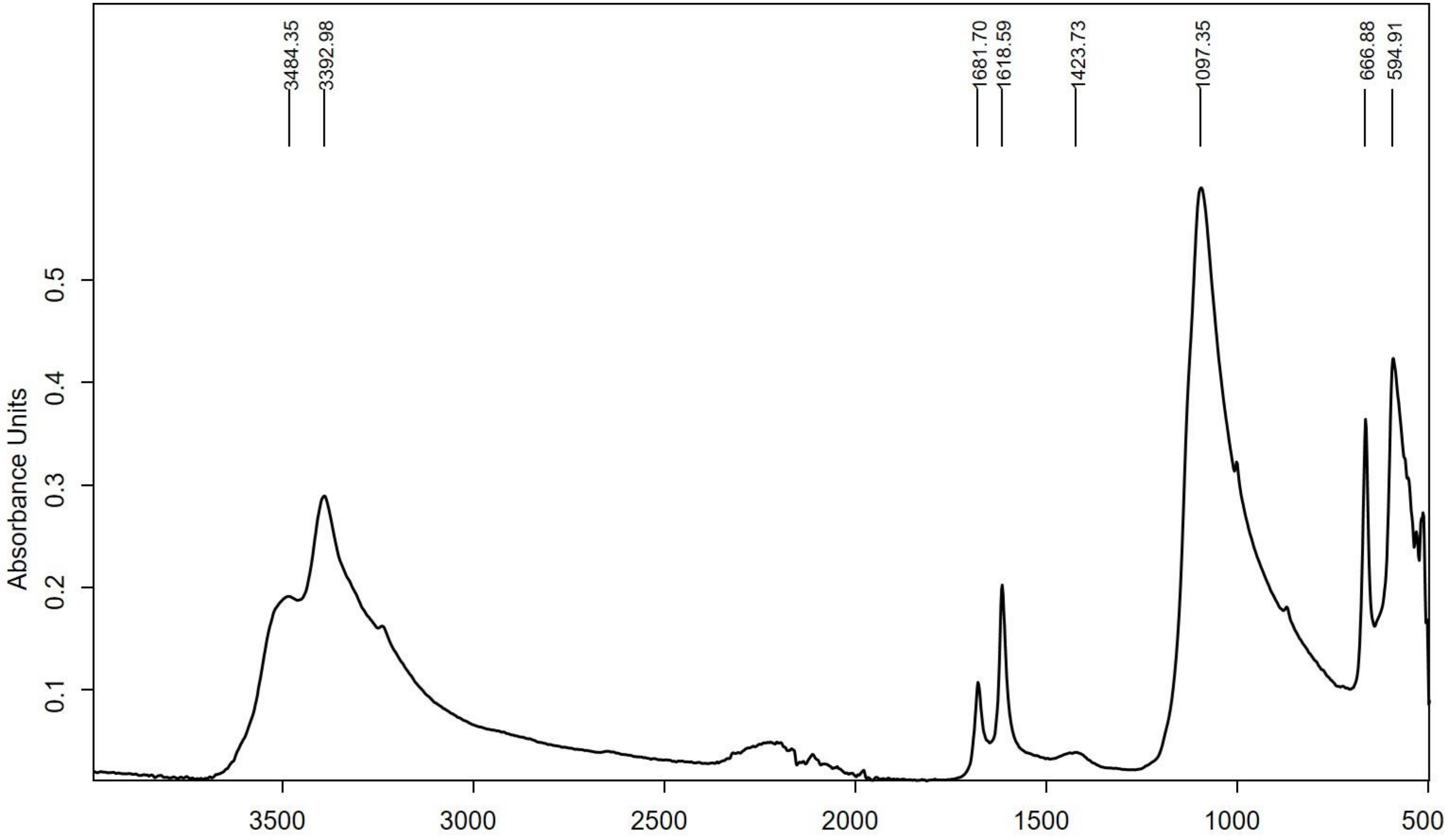

Figure S20.- IR spectrum obtained in the paint layers of sample J-3.

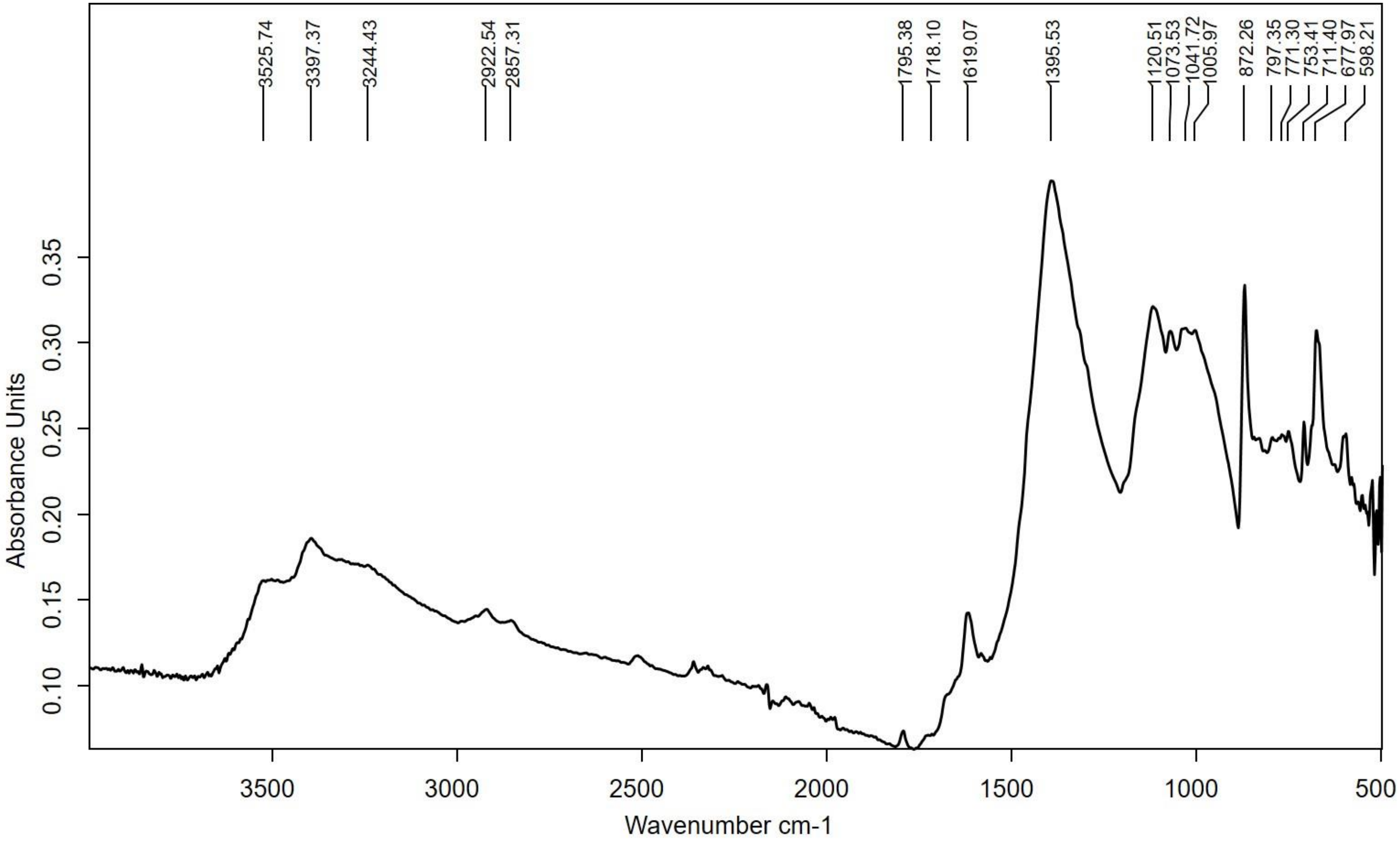

Figure S21.- Detail of the previous IR spectrum obtained in the amide I region of the paint layer in sample J-3.

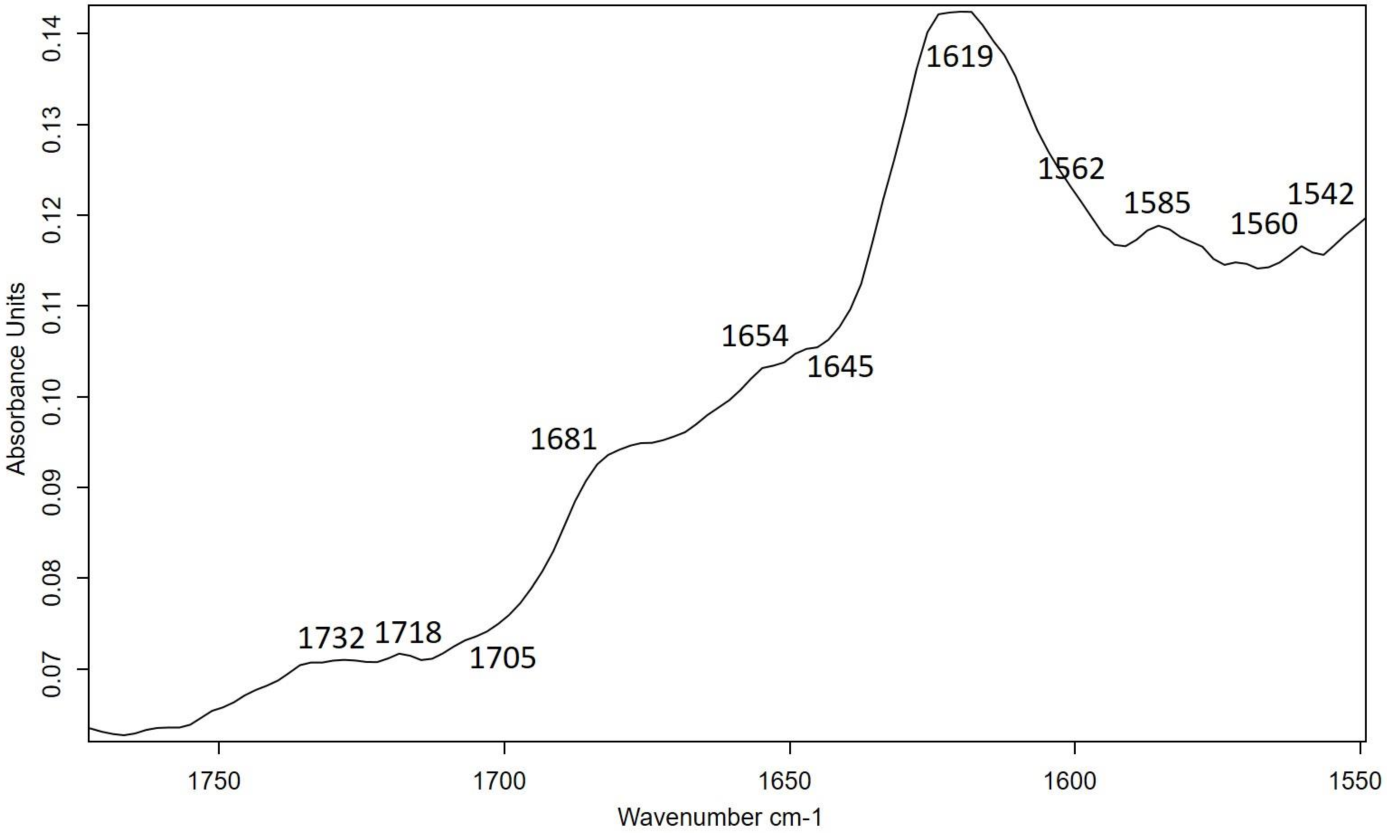

Figure S22.- Detail of the previous IR spectrum obtained in the 1200-550 cm^{-1} region of the paint layer in sample J-3.

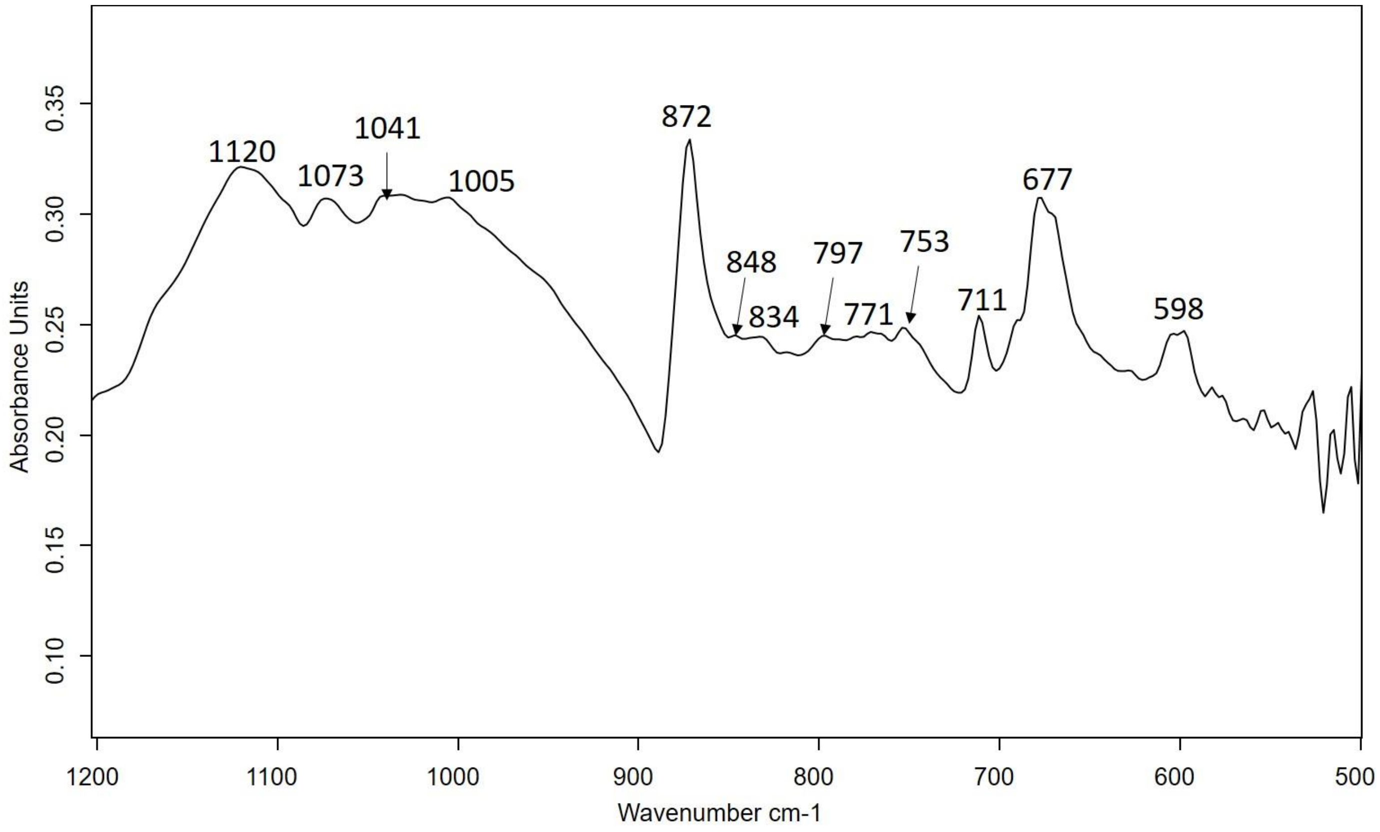

Figure S23.- IR spectrum obtained in the ground of sample J-3.

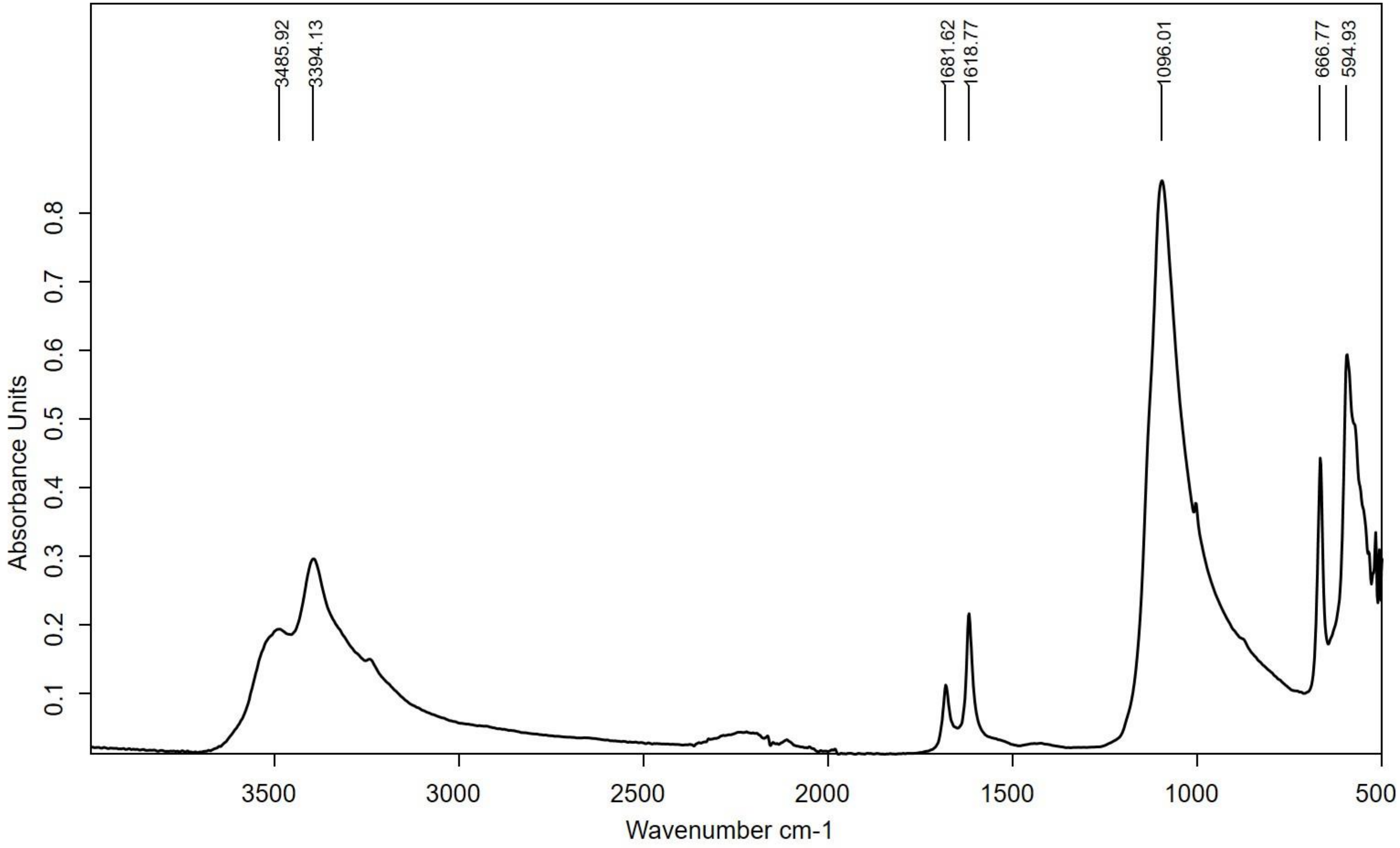

Figure S24.- IR spectrum obtained in the ground of sample J-4.

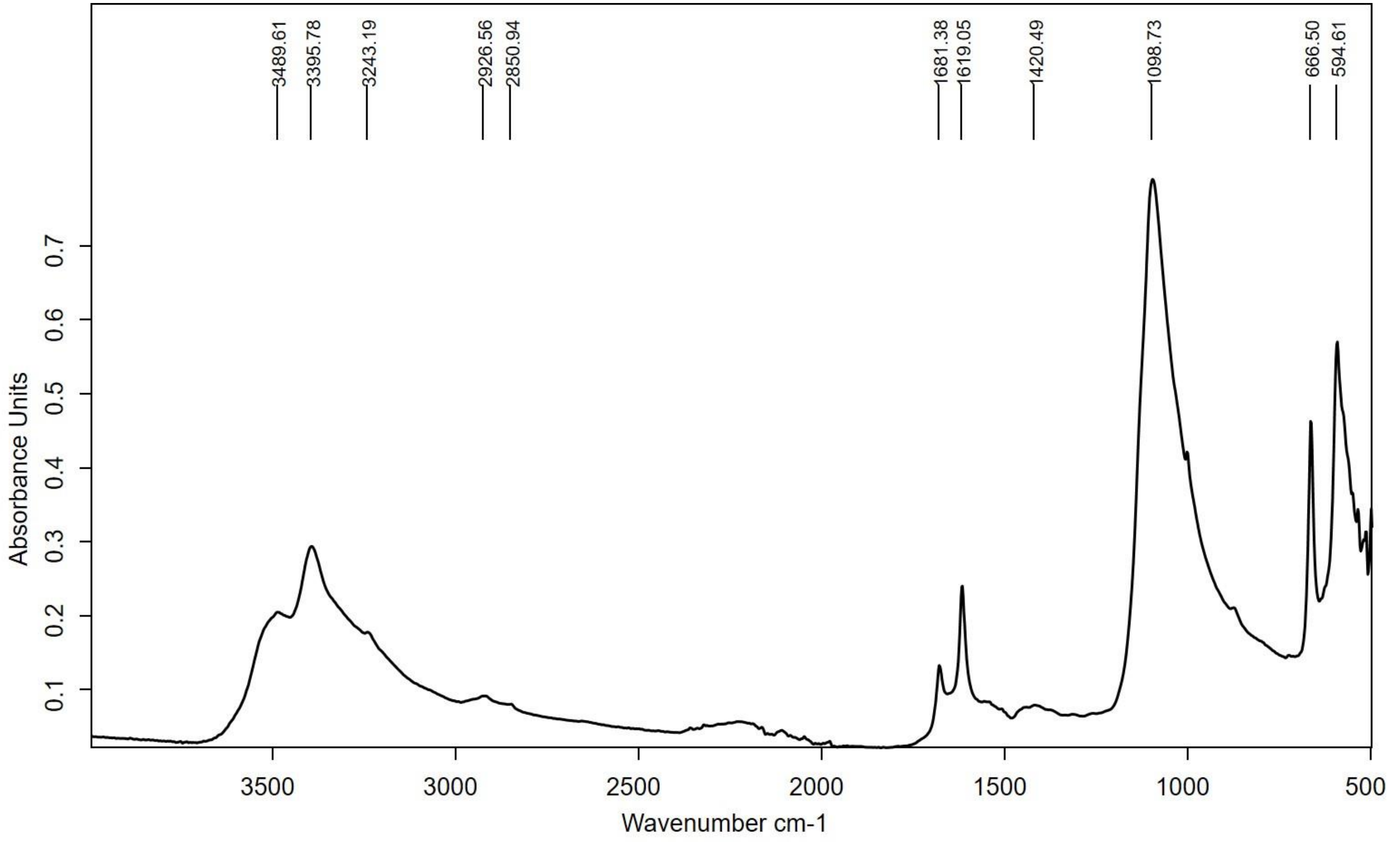

Figure S25.- IR spectrum obtained in the paint layers of sample K-4.

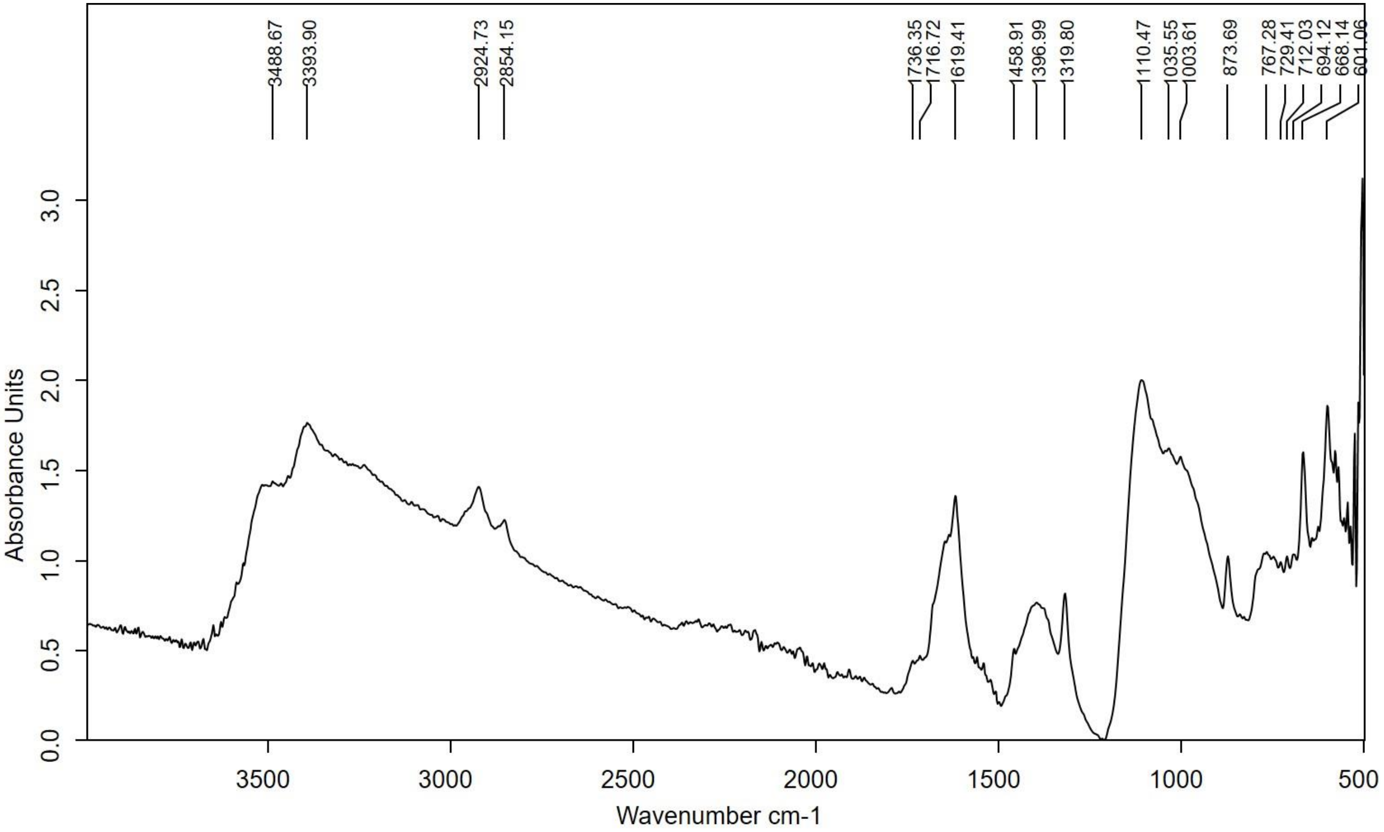

Figure S26.- Detail of the previous IR spectrum obtained in the amide I region of the paint layer in sample K-4.

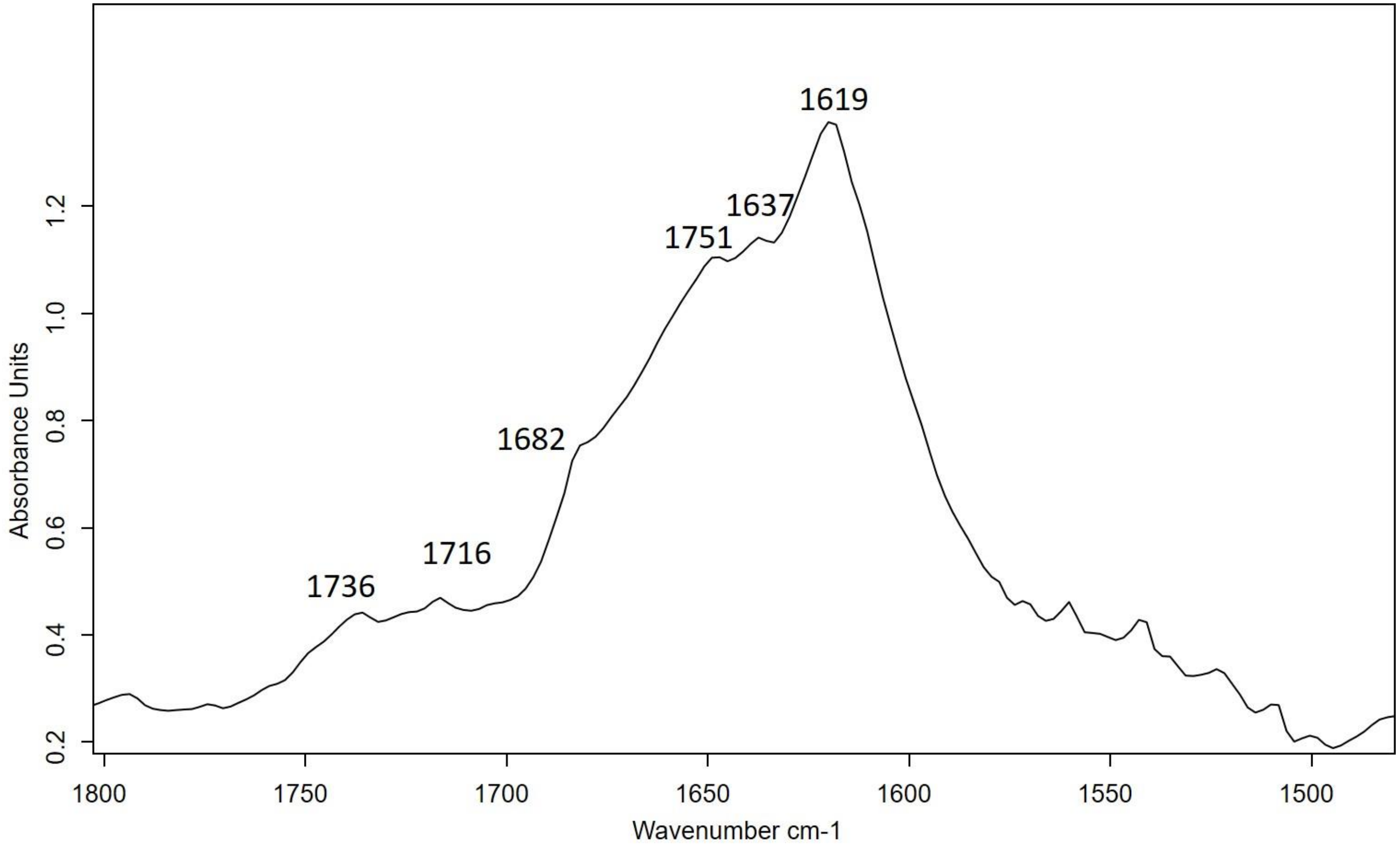

Figure S27.- IR spectrum obtained in the ground of sample K-4.

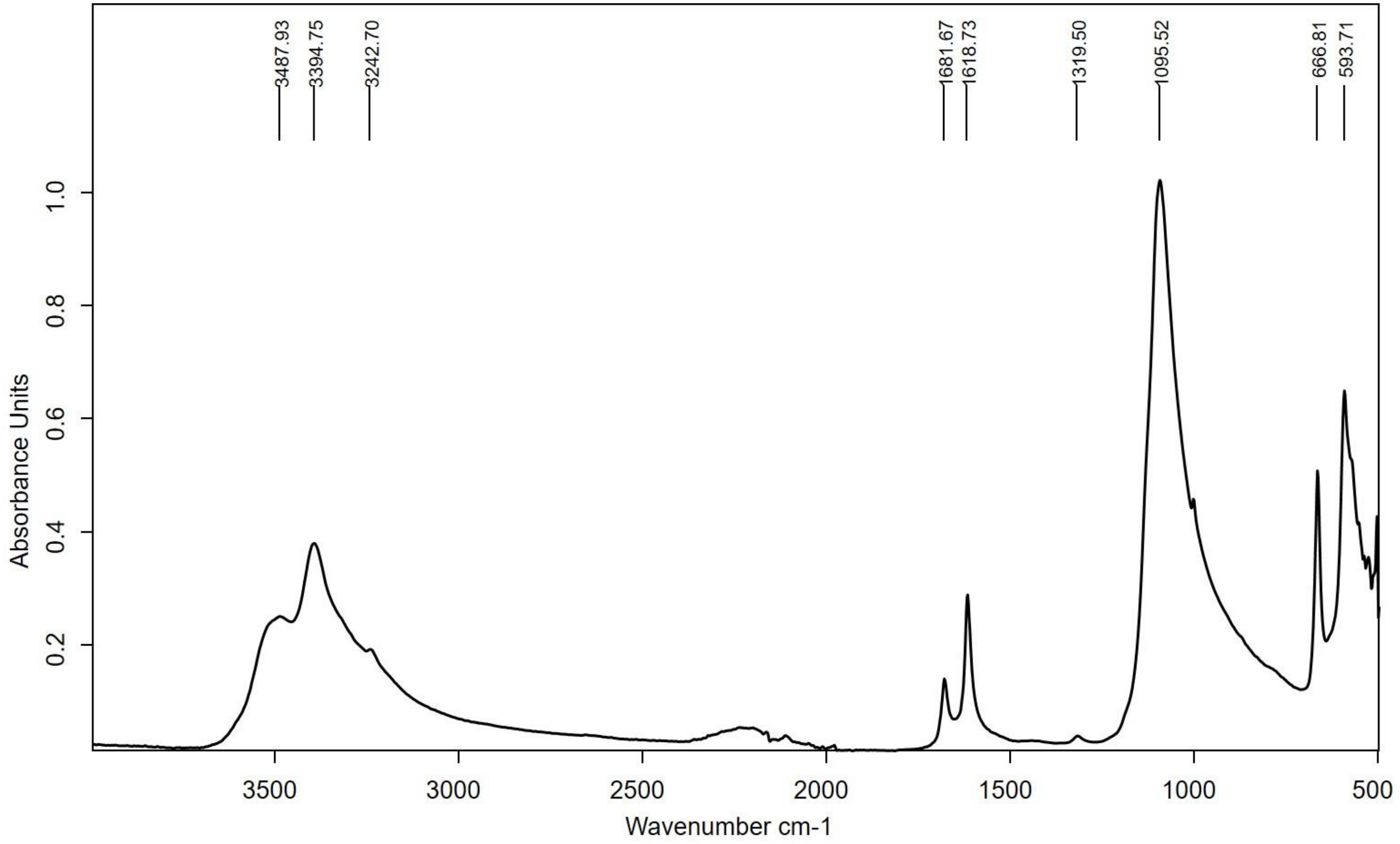

Table S3: Secondary electron images of trenches and X-ray spectra and elemental composition.

SAMPLE C-2

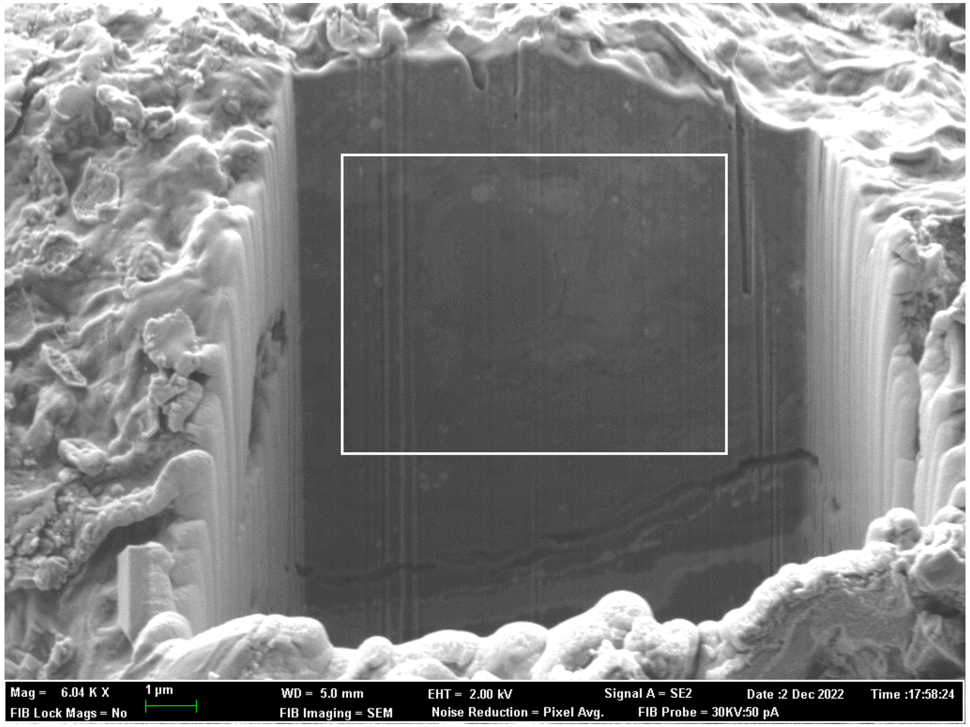

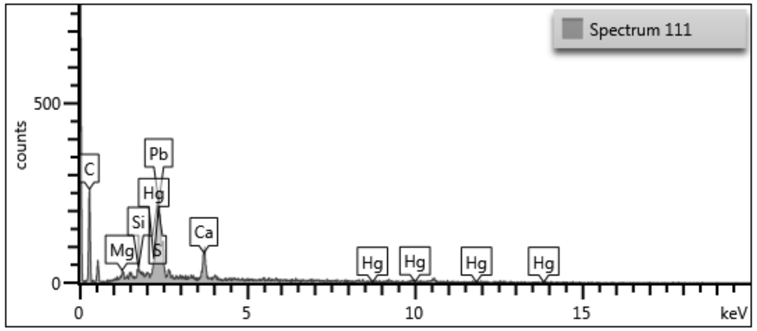

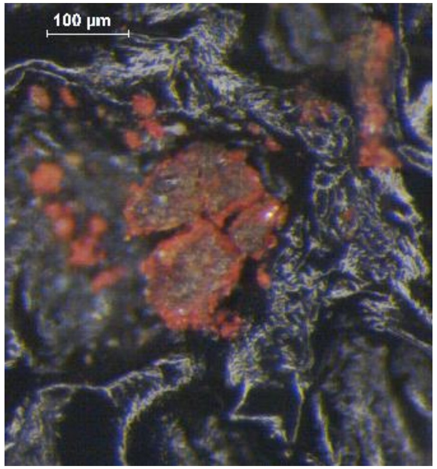

Element	Wt%
O	22,06
Mg	1,21
Si	1,33
S	6,93
Ca	12,59
Hg	11,21
Pb	44,67
Total:	100

SAMPLE C-5

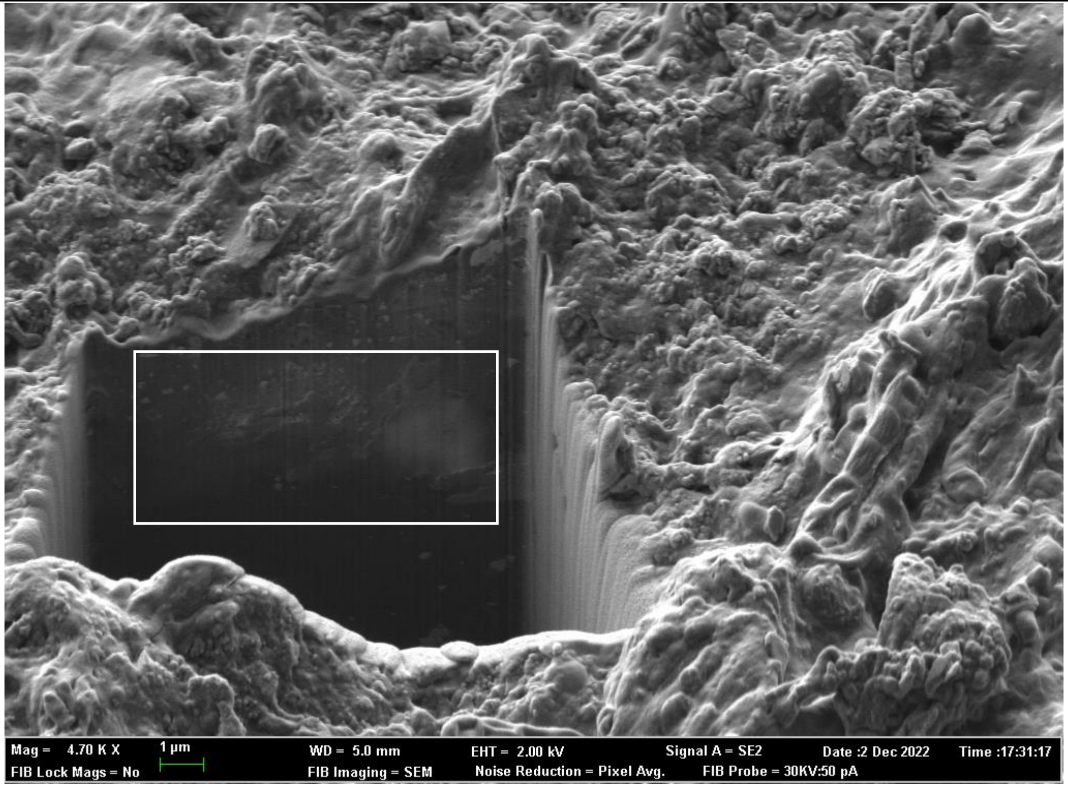

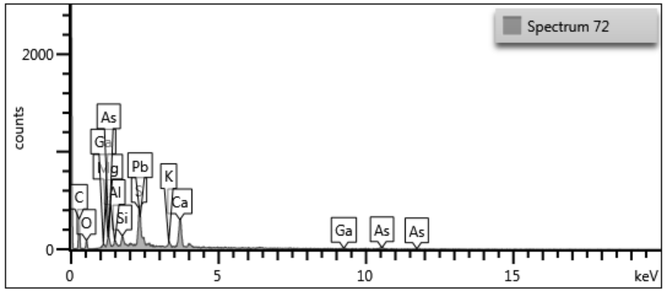

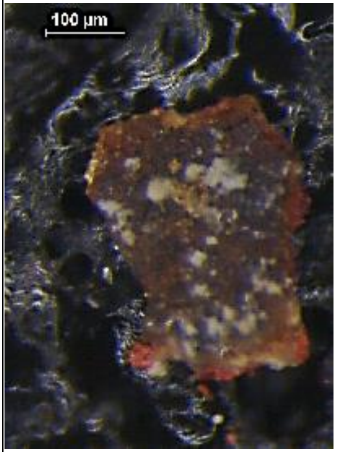

Element	Wt%
O	37,67
Mg	2,63
Al	1,53
Si	2,95
S	13,02
K	2,74
Ca	20,22
Ga	3,96
As	2,29
Pb	12,99
Total:	100

SAMPLE D-1

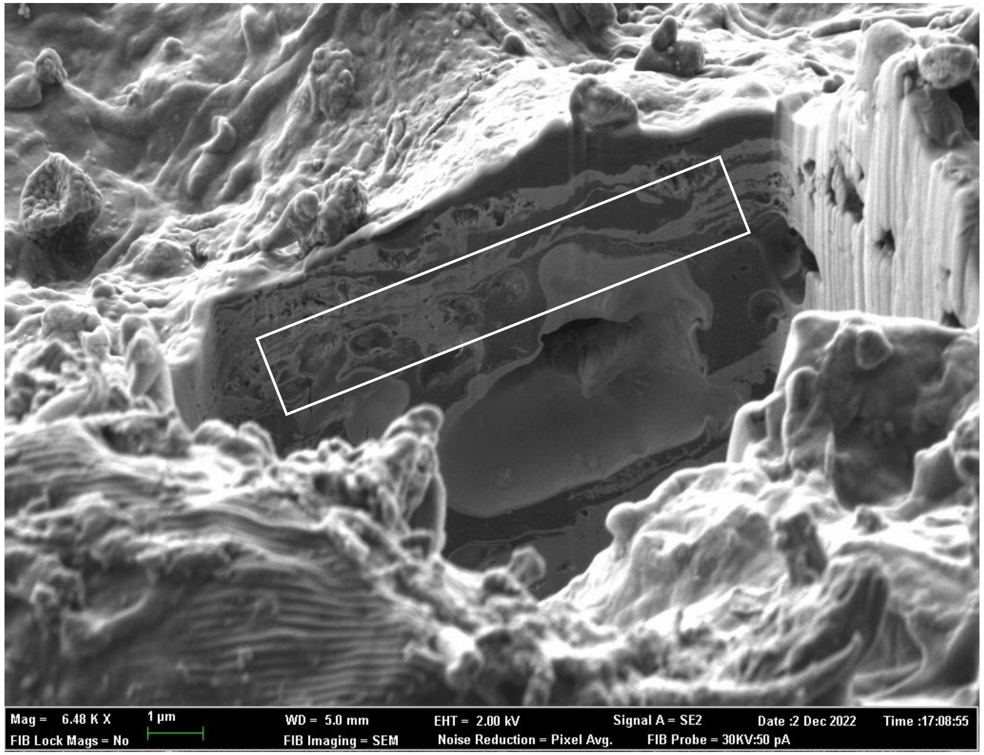

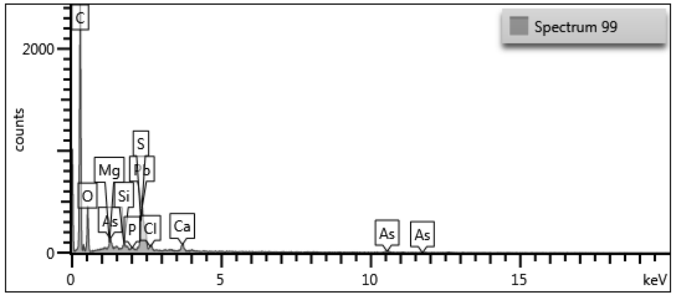

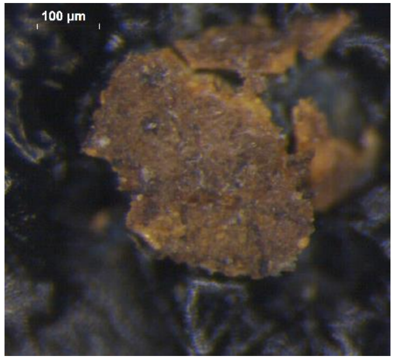

Element	Wt%
O	19,38
Mg	1,52
Si	1,82
P	0,94
S	4,27
Cl	2,43
Ca	6,3
As	5,34
Pb	57,99
Total:	100

SAMPLE F-1

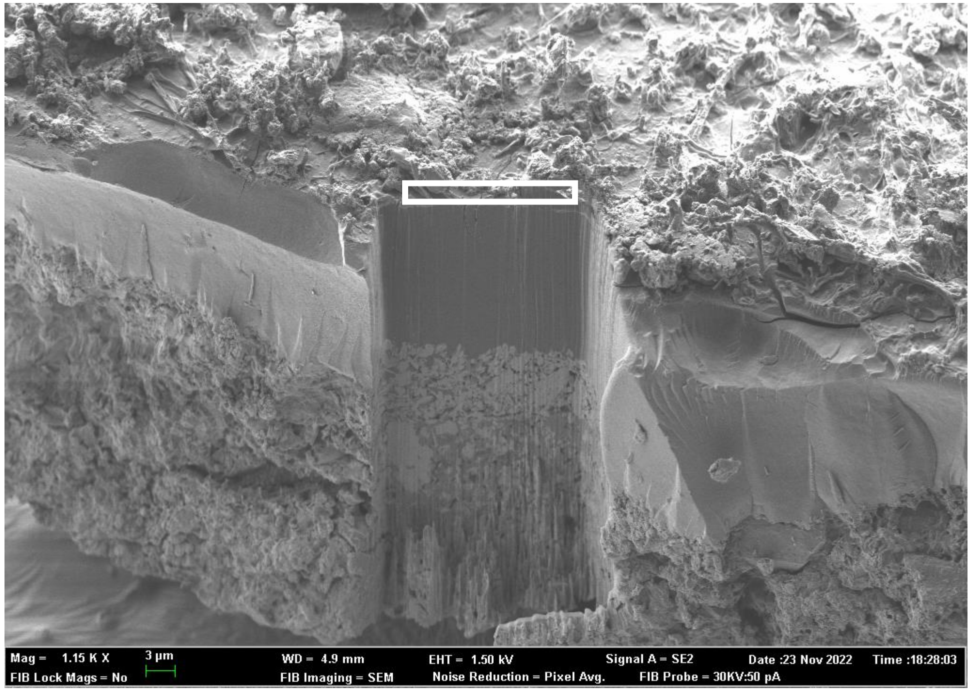

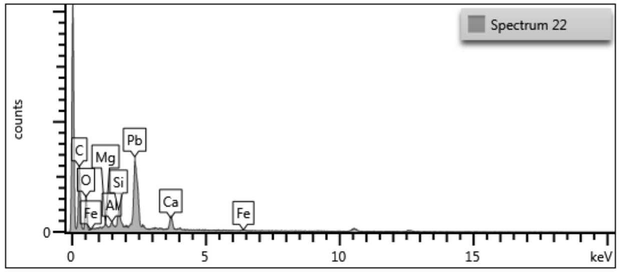

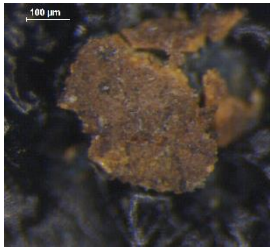

Element	Wt%
O	15,24
Mg	0,93
Al	1,19
Si	4,21
Ca	7,79
Fe	0,99
Pb	69,65
Total:	100

SAMPLE J-4

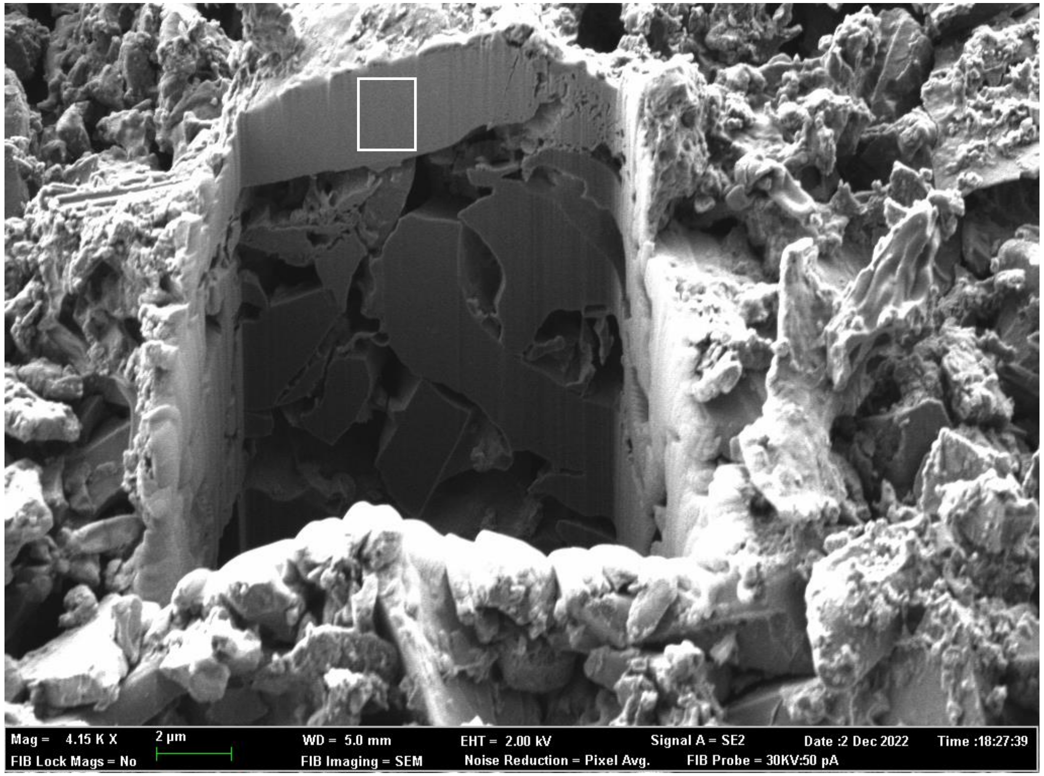

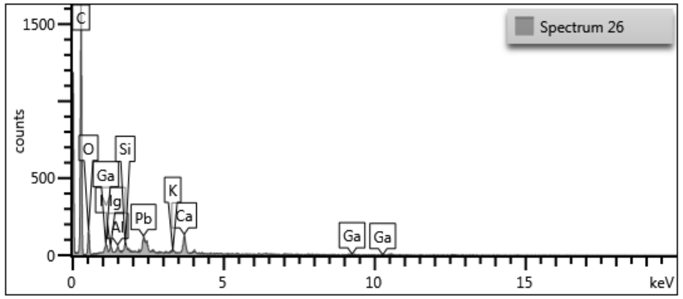

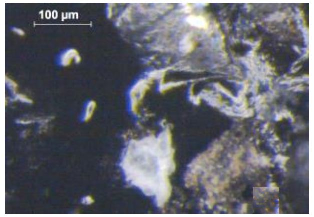

Element	Wt%
O	22,9
Mg	3,03
Al	3,07
Si	3,95
K	2,56
Ca	22,98
Ga	2,91
Pb	38,6
Total:	100

SAMPLE K-4

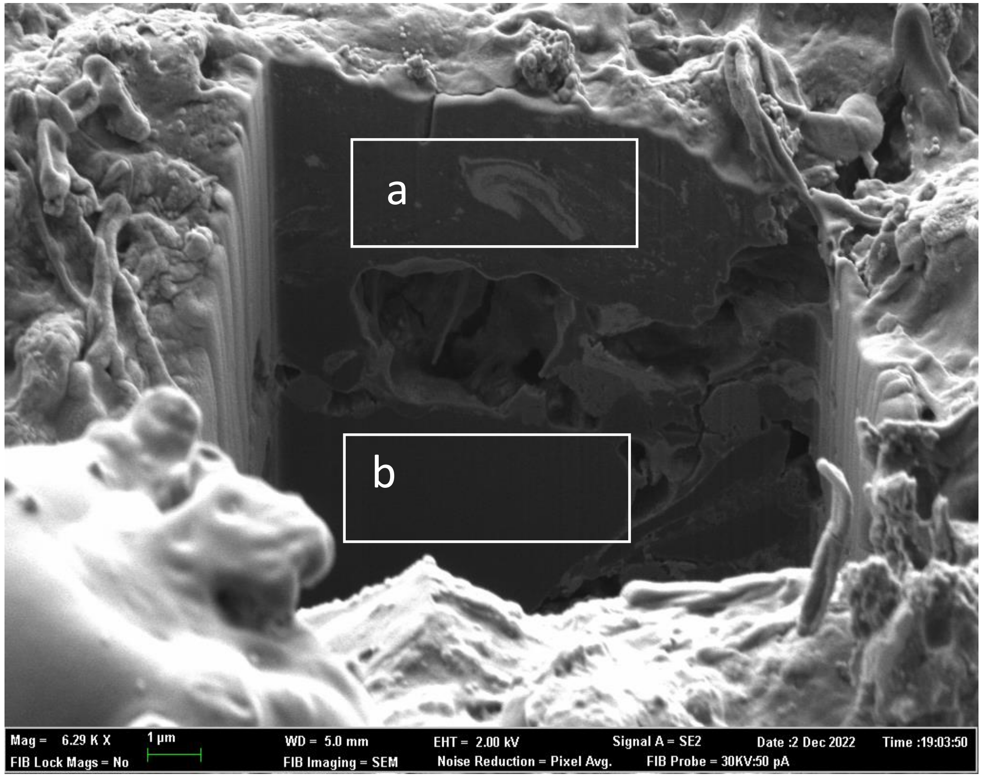

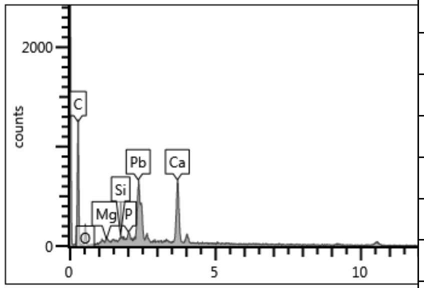

Area a	
Element	Wt%
O	18,67
Mg	0,68
Si	0,9
P	1,58
Ca	28,3
Pb	49,87
Total:	100

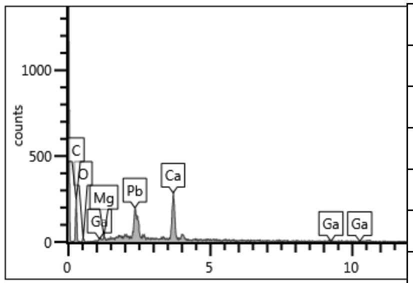

Area b	
Element	Wt%
O	18,86
Mg	1,23
Ca	35,29
Ga	1,93
Pb	42,68
Total:	100

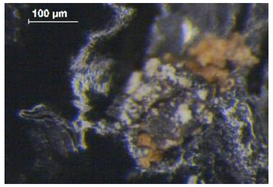

Table S4.- Experimental working conditions of FESEM-EDX

Working conditions<br>A FESEM Zeiss (Orsay Physics Kleindiek Oxford Instruments) model Auriga compact equipment was used for the FESEM-EDX examination. The X-ray microanalysis was performed using an Oxford-X Max X-ray microanalysis system coupled to the FESEM.<br>Operation conditions in the FESEM-EDX: A voltage of 20 kV, a current beam of 3.76-3.83 μA, resolution of 127 eV at 5.9 keV. X-ray detector operates at a working distance of 6-7 mm. The electron beam was always disposed perpendicularly to the polished surface of the cross-sections.<br>X-ray spectra acquisition conditions were: dead time 20 %, minimum number of counts acquired 2000, live time acquisition mode with an acquisition time of 100 s, process time of 5 s, number of channels automatically selected by the instrument that provided a channel width of 10 eV. Pulse pileup correction was active, which automatically performs deconvolution of overlapped peaks.<br>As samples are poor conductor, they were carbon coated for avoiding localized charging and any resulting distortion or reflection of the electron beam.<br>Software Aztec (Orsay Physics Kleindiek Oxford Instruments) has been used for controlling the acquisition of digital images and X-ray spectra.<br>Images were acquired with the backscattered electron detector. A scansize of 1024, dwell time of 34 μs and frame time of 26.739 s were the image acquisition conditions.<br>The software Aztec automatically considers the C sputtering treatment and automatically re-calculates the intensity of the emission line of the C peak according to a calculated thickness of the external layer of C formed by the sputtering of 10 μm and density of 2.25 g cm^{-3}. Therefore, presence of C-containing minerals and organic matter was established from the rest of analytical techniques applied in this research.<br>Certificate reference standard materials used for calibrating the instrument:<br>C: $CaCO_3$; O: SiO_2; Na: Albite; Mg: MgO; Al: Al_2O_3; Si: SiO_2; P: GaP; S: FeS_2; Cl: KCl; K: MAD-10 Feldspar; Ca: wollastonite; Ti: Ti; Mn: Mn; Fe: Fe; Cu: Cu; Pb: PbF_2; As: InAs; Co: Co; Sb: Sb; Sn: Sn.
Qualitative measurements:<br>LOD: a theoretical averaged LOD for SEM–EDS measurements has been established in 0.08 wt% (Reed, 1996). Nevertheless, a common procedure for a more accurate calculation of the LODs in the FESEM and SEM from experimental measurements procedures (Veritá et al, 1994). According to this last method the averaged LOD value calculated from the experimental X-ray spectra obtained for the set of elements analyzed are in the range 0.07 - 0.1 wt%.
Quantitative measurements:<br>The standard deviation of the wt% values of the different elements is calculated by the Aztec software after applying the ZAF method of correction of interelemental effects on the intensity values for each element in each X-ray spectrum. Values obtained are in good agreement with those reported in similar studies of archaeological glass and ceramic materials (Kuisma-Kursula, 2000).<br>Accuracy of the FESEM-EDX instrument and applied method is calculated from NIST clay standard reference material (SRM 679-Brick clay) and NIST glass standard reference material (SRM 620-soda-lime glass) for considering the effect of the state of the sample (irregular and multi-mineralogical phase powder or flat and homogeneous amorphous material) in the accuracy provided by the analytical method. The values provided in the table thereafter correspond to the relative error, expressed in percentage ((experimental observed composition – certified composition) x)/certified composition)) x100%. Results obtained are similar to those reported by other authors previously (Kuisma-Kursula, 2000).

**NIST brick-clay SRM 679 Brick clay powder**			**NIST Glass SRM 620 Soda-lime glass platelets (35x35x3) mm**		
**Element**	**certified (wt %)**	**RD (%)**	**Oxide**	**certified (oxide wt %)**	**RD (%)**
Al	11.01	3	SiO_2	72.08	2
Ba	0.0432	145	Al_2O_3	1.80	5

Ca	0.1628	45	Fe_2O_3	0.043	87
Ce	105*	n.d.	TiO_2	0.018	110
Cs	9.2*	n.d.	CaO	7,11	2
Cr	109.7*	n.d.	MgO	3,69	3
Co	26*	n.d.	K_2O	0,41	6
Cu	1.9*	n.d.	Na_2O	14,39	2
Hf	4.6*	n.d.	As_2O_3	0,056	75
Li	71.7*	n.d.			
Mg	0.7552	5			
Mn	1730*	9			
P	0.075	46			
K	2.43	7			
Rb	190*	n.d.			
Sc	22.5*	n.d.			
Si	24.34	2			
Na	0,1304	4			
Sr	73.4*	n.d.			
Th	14*	n.d.			
Ti	0.577	5			
Zn	150*	n.d.			
Fe	9.05	3			
* value is in mg kg^{-1}					
n.d.: not detected					

References

Reed, S.J.B., (1996) Electron Microbe Analysis and Scanning Electron Microscopy in Geology. Cambridge University Press, Cambridge.

Veritá, M., Basso, R., Wypyski, M.T., Koestler, R.J., (1994), X-ray microanalysis of ancient glassy materials: a comparative study of wavelength dispersive and energy dispersive techniques, Archaeometry, 36, 241-25.

Kuisma-Kursula, P., (2000), Accuracy, Precision and Detection Limits of SEM–WDS, SEM–EDS and PIXE in the Multi-Elemental Analysis of Medieval Glass, X-Ray Spectrometry, 29, 111–118.
